# Quantitative conversion of biomass in giant DNA virus infection

**DOI:** 10.1038/s41598-021-83547-9

**Published:** 2021-03-03

**Authors:** Mikael Kördel, Martin Svenda, Hemanth K. N. Reddy, Emelie Fogelqvist, Komang G. Y. Arsana, Bejan Hamawandi, Muhammet S. Toprak, Hans M. Hertz, Jonas A. Sellberg

**Affiliations:** 1 Biomedical and X-Ray Physics, Department of Applied Physics, AlbaNova University Center, KTH Royal Institute of Technology, 106 91 Stockholm, Sweden; 2 Laboratory of Molecular Biophysics, Department of Cell and Molecular Biology, Uppsala University, Husargatan 3 (Box 596), 751 24 Uppsala, Sweden

**Keywords:** Viral host response, Microscopy, Microbiology techniques

## Abstract

Bioconversion of organic materials is the foundation of many applications in chemical engineering, microbiology and biochemistry. Herein, we introduce a new methodology to quantitatively determine conversion of biomass in viral infections while simultaneously imaging morphological changes of the host cell. As proof of concept, the viral replication of an unidentified giant DNA virus and the cellular response of an amoebal host are studied using soft X-ray microscopy, titration dilution measurements and thermal gravimetric analysis. We find that virions produced inside the cell are visible from 18 h post infection and their numbers increase gradually to a burst size of 280–660 virions. Due to the large size of the virion and its strong X-ray absorption contrast, we estimate that the burst size corresponds to a conversion of 6–12% of carbonaceous biomass from amoebal host to virus. The occurrence of virion production correlates with the appearance of a possible viral factory and morphological changes in the phagosomes and contractile vacuole complex of the amoeba, whereas the nucleus and nucleolus appear unaffected throughout most of the replication cycle.

## Introduction

Detailed knowledge of the replication cycle of viruses in host cells is essential for understanding viral infections and developing virotherapy^1^. Although the mechanism of infection varies greatly among virus families, the end result is usually a burst of viruses released from the infected—and soon dead—cell upon lysis. The newly produced virions can then repeat the infection process inside a new, healthy cell. Investigations of the replication process typically involve high-resolution microscopy, providing important qualitative information. For quantitative data, e.g. number of virus particles making up the viral burst, end-point dilution titration measurements are commonly performed^2^. In order to estimate the transfer of carbonaceous biomass from the host cell to the virus, the burst data must be complemented with assumptions of carbon density in the virus and host cell. In the present paper we introduce an emerging high-resolution microscopy method, water-window X-ray microscopy, and show that it allows quantitative imaging over the full infection cycle of viral particles large enough to be identified in the cell. Furthermore, we complement the classical burst size measurements with thermal gravimetric analysis (TGA) to provide a more accurate quantitative assessment of conversion of biomass. As proof of concept, we apply the methods to an unidentified giant DNA virus infecting *Acanthamoeba castellanii* and compare the measured conversion with the morphological changes of the host cell post infection.

Viral replication in host cells is usually studied by high-resolution histology of ultrathin (usually 50–300 nm) tissue sections using transmission electron microscopy (TEM). Unfortunately, the embedding process risks altering the sample and the thin sectioning makes quantitative studies difficult. Focused ion beam (FIB) milling can be used on cryo-frozen specimens to circumvent the embedding process, which was combined with electron tomography on vesicles containing Herpes virus particles at the nuclear membrane^3^. Alternatively, FIB combined with scanning electron microscopy (SEM) has been applied to study viral replication of *Paramecium bursaria chlorella* virus 1^4^, as well as compartmentalization of the *Pseudomonas* bacteriophage 201φ2-1 within its host^5^. Although FIB-SEM can probe a much larger volume than TEM, the time-consuming acquisition of the full image stack prohibits quantitative studies of viral replication in large cohorts of cells.

Soft X-ray microscopy makes it possible to quantitatively study viral replication in the near-native state of the intact host cell, since it enables high-resolution imaging and requires neither sectioning nor staining^6^. The high contrast between biomolecules—containing mostly carbon and nitrogen—and water—containing mostly oxygen—is utilized in the so-called ‘water window’ between the carbon K-edge at *λ* = 4.3 nm and oxygen K-edge at *λ* = 2.3 nm^7^. Despite these advantages, few studies have so far probed viral replication using soft X-rays. Soft X-ray cryo-tomography has been used to image cells infected by Hepatitis C virus^8^, Pseudorabies virus^9^ and Vaccinia virus^10^, out of which only Vaccinia viral particles could be detected inside the cells. Chichon et al*.*^10^ were able to detect mature and immature Vaccinia viral particles, where the latter had lower absorption contrast. In addition, they reported slightly modified mitochondria and identified viral factories as parts of the cell with higher concentration of viral particles.

In the present study we quantitatively investigate the viral replication of a giant DNA virus from infection to cell death. Giant viruses, defined herein as those exhibiting viral particles with a smallest dimension exceeding 0.3 µm^11^, infect protozoa of the amoebozoan genus *Acanthamoeba* and have been classified into four families^11^: *Mimiviridae*^12,13^, *Pandoraviridae*^14,15^, *Pithoviridae*^16–20^ and the unclassified genus Mollivirus^21^. They all have double-stranded DNA genomes larger than 500 kbp and can either be exclusively cytoplasmic or recruit functions from the host nucleus for the earliest phases of their infectious cycles^11^.

The replication process of the unidentified isolate is studied using the Stockholm laboratory X-ray microscope, an instrument which does not require large-scale infrastructure and provides high-resolution imaging of the intact cell-virus system in its near-native state. The microscope provides high carbon-to-water contrast without any staining at a half-period resolution of 35 nm^22^. Subsequent image analysis provides quantitative assessment of viral replication as well as qualitative analysis of cell morphology. The intrinsic carbon contrast of the water-window X-ray region provides a path to quantitative determination of carbonaceous biomass converted from cellular material to virus. The microscopy results are supported by quantitative end-point measurements based on dilution titrations combined with TGA on amoeba and virus. The implications of the quantitative findings are discussed in terms of host metabolism and energy budget.

## Results

### Virus isolation and morphology

**Figure 1:**
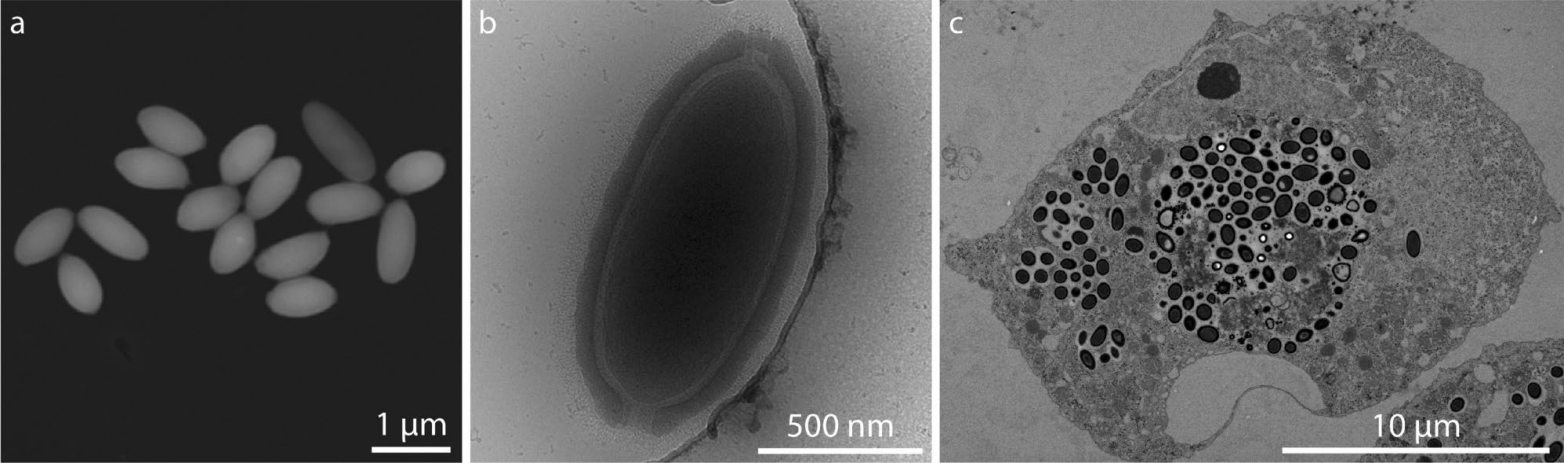
Electron microscopy of virions of Lurbovirus. (**a**) Scanning electron micrograph of isolated virions of Lurbovirus using a scanning transmission electron microscope (STEM) detector in high-angle annular dark-field mode. (**b**) Transmission electron micrograph of a cryo-frozen virion of Lurbovirus. The double cork-like structures indicate morphological similarity to Cedratviruses. (**c**) Transmission electron micrograph of a sectioned, stained, resin-embedded, virus-infected amoeba cell imaged 10 hpi (at 32 °C in medium containing 20 mM glucose). Scale bars are 1 µm, 500 nm and 10 µm, respectively.

A giant virus (Fig. 1a) was isolated from a soil sample collected from the banks of a small stream southwest of Uppsala, Sweden. The isolated virion in Fig. 1b has an ovoid shape, with dimensions of 0.59 × 1.25 µm^2^. By morphological similarity, this unidentified virus is likely a member of the *Pithoviridae* family, possibly a Cedratvirus^18–20^, because all observed particles contained double cork-like structures (Fig. 1b), one at each apex. We refer to the virus herein as Lurbovirus based on the place of isolation. Full genome sequencing and classification is ongoing and will be published separately.

Like other members of the *Pithoviridae* family, TEM reveals that the Lurbovirus produces virions in the cytoplasm of the amoeba while the nucleus often remains intact (Fig. 1c). Furthermore, the virions are frequently produced inside membrane-bound vesicles at the periphery of a viral factory (Supplementary Information).

### X-ray microscopy of virus infections

**Figure 2:**
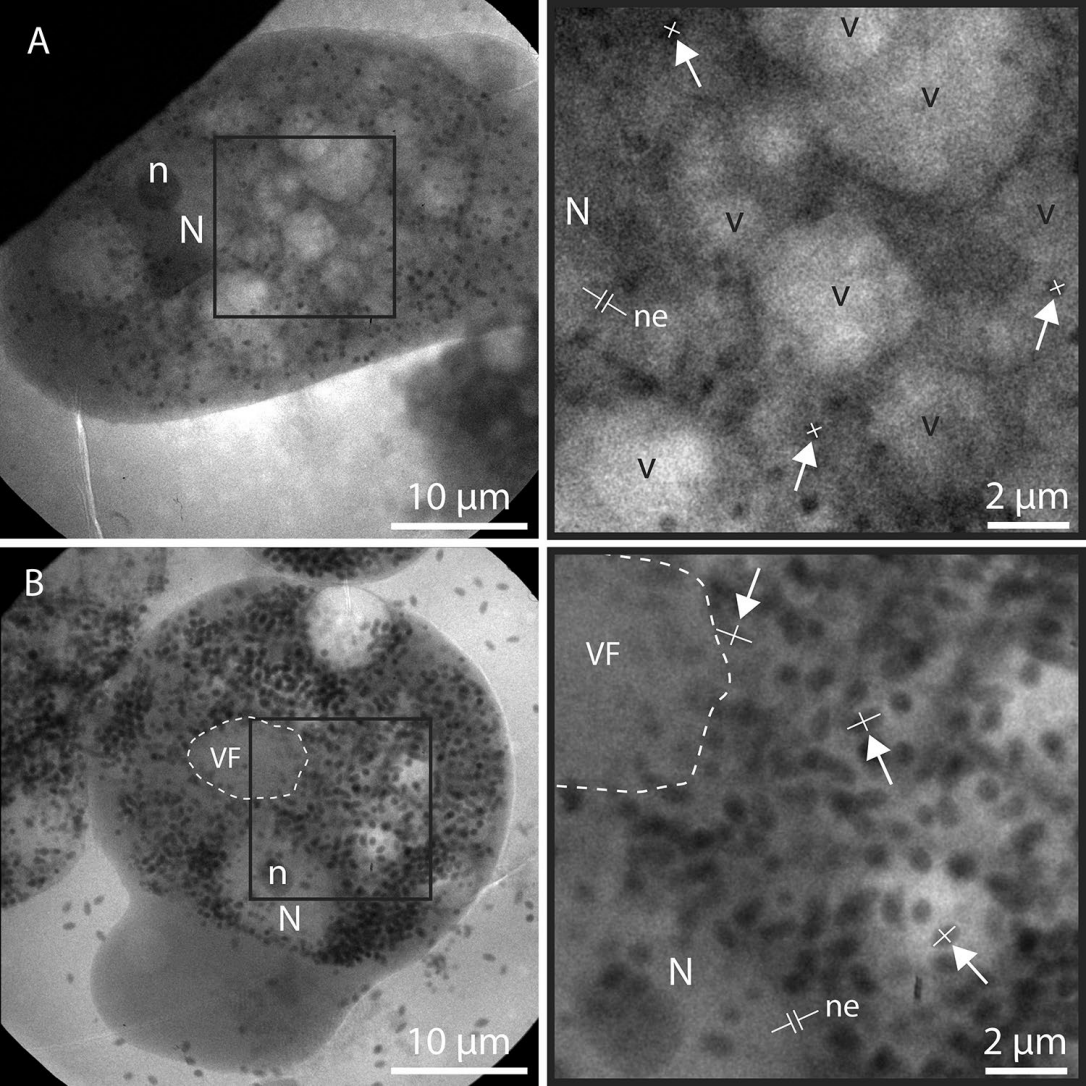
Laboratory full-field water-window cryo-microscopy of non-infected and infected amoeba. X-ray micrographs of a typical (**a**) non-infected amoeba (n = 14) and (**b**) infected amoeba imaged 54 hpi (n = 5). The images to the right show enlargements of the square insets. The appearance of numerous large (~ 1-µm length) ovoid virions in the infected cell clearly distinguishes it from the non-infected cell, which contains smaller (typically < 500 nm) and more circular objects, probably mitochondria or storage granules. A possible viral factory (VF) in the vicinity of the nucleus is indicated by the dashed white line. In order to clearly show the difference between the virions and the smaller X-ray absorbing objects, three examples in each image have been marked by arrows and a white cross indicating their length and width. Another striking difference is the typical vacuolated structure (v) of the non-infected cell, which is less prominent in the infected cell. Note that the nucleus (N) including the darker nucleolus (n) and the nuclear envelope (ne) appears to be intact in both cells. Scale bars are 10 µm in the whole images and 2 µm in the enlargements.

Figure 2 shows a detailed comparison between a typical non-infected healthy amoeba (*Acanthamoeba castellanii*) (Fig. 2a) and a Lurbovirus-infected amoeba (Fig. 2b) imaged 54 h post infection (hpi), for which the infection has fully matured without obvious signs of lysis. In both cases, the samples were cryo-fixed and imaged using the Stockholm laboratory X-ray microscope. The infection process was performed as detailed in “Materials and methods”. It should be mentioned that in our imaging experiments the cell media contained no glucose and the cells were kept at room temperature during the whole process. This results in a prolonged infection process as compared to other published results for related viruses^11,18–20^.

The non-infected cell has a vacuolated structure (bright circles few µm in diameter) (Fig. 2a), which is typical for this kind of amoeba^23^. Small (< 500 nm) and close-to circular X-ray absorbing objects, probably mitochondria or storage granules^24,25^, are distributed throughout the cell. The nucleus can be easily identified due to the X-ray absorbing nucleolus and the absence of vacuoles and other cellular structures. Even the nuclear envelope can be seen (Fig. 2a,b), albeit not completely resolved.

The extensive viral production in the infected cell (Fig. 2b) is evident from the numerous ovoid virions—typically ~ 1 µm long—in practically all parts of the cell, except for the nucleus, which remains intact. The virions are in most cases easily distinguishable from the other small, X-ray absorbing objects in the healthy cells due to their larger size and ovoid shape. The outline of a possible viral factory in the vicinity of the nucleus is indicated by the dashed line. This outlined area is almost clear of any X-ray absorbing objects, except for some low-absorbing viral material and particles along its periphery, which is consistent with TEM of Lurbovirus (Supplementary Information) and previously published TEM images of related viruses^16,19,20^.

### Progression of viral replication cycle

**Figure 3:**
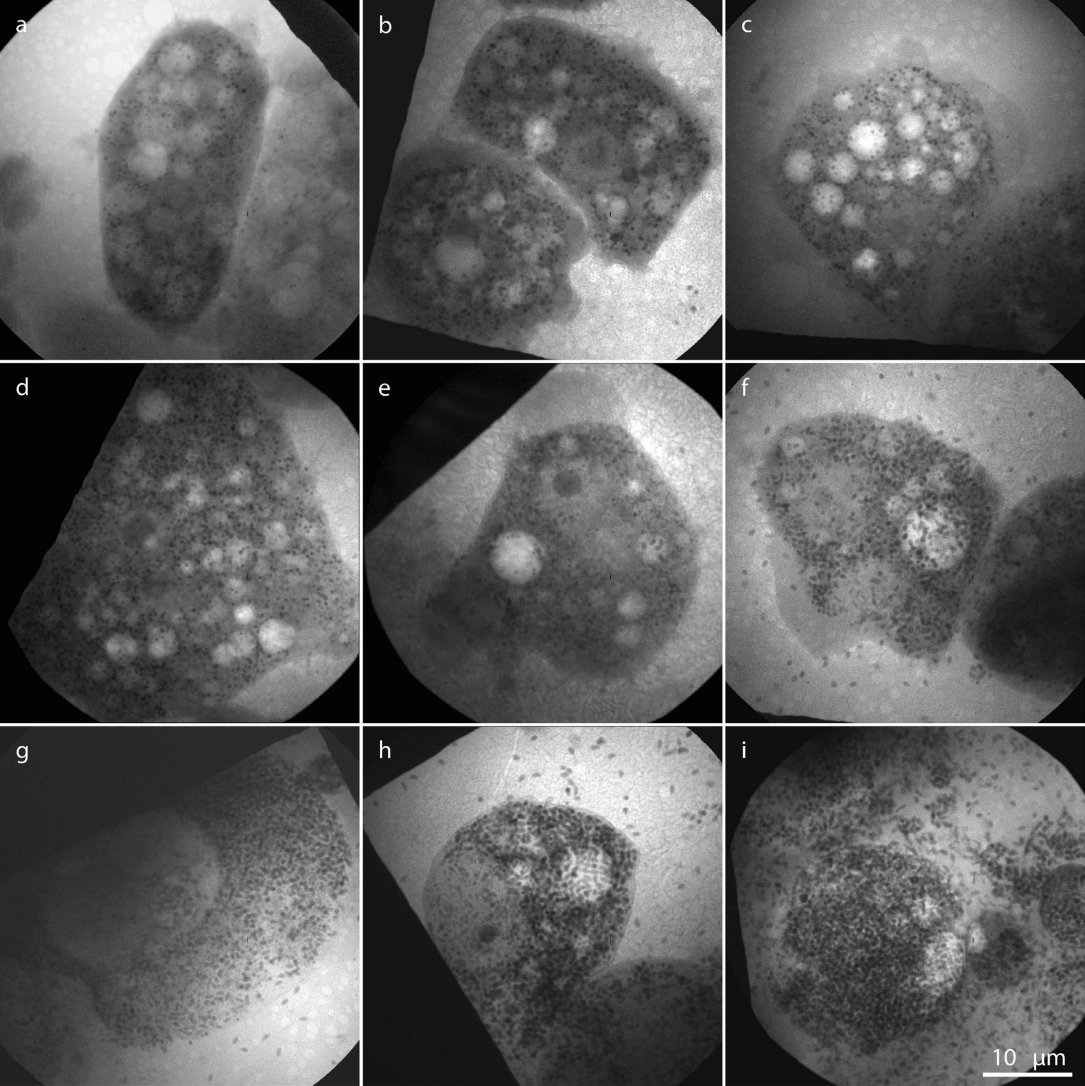
Virus infection series, ranging from non-infected cells to cells 70 hpi, visualized using laboratory cryogenic X-ray microscopy. (**a**) Non-infected amoeba (n = 5) that is included for comparison and has been treated identically as the infected amoebae 30 hpi aside from not adding the virus. The infection times in the displayed images are (**b**) 6 h (n = 5), (**c**) 12 h (n = 6), (**d**) 15 h (n = 5), (**e**) 18 h (n = 6), (**f**) 24 h (n = 5), (**g**) 30 h (n = 5), (**h**) 54 h (n = 5) and (**i**) 70 h (n = 4), respectively. In the images up to 15 hpi (**a**–**d**) no clearly distinguishable virions were detected. At 18 hpi (**e**) a few virions can be seen, e.g. in a vacuole in the right part of the cell as well as to the lower left of the nucleus. At longer infection times (**f**–**i**) the virions take up an increasing part of the cell volume, as well as the surrounding medium, until they appear densely packed throughout the cell. Possible viral factories, like the one marked in Fig. 2, can be seen close to the nuclei at 18–30 hpi (**e**–**g**). The vacuolated structure of the healthy cells seems to disappear as the production of virions takes off, while the cell nucleus can be seen in all images up to 54 hpi (**a**–**h**). Scale bar is 10 µm and valid for all images.

Figure 3 shows laboratory cryogenic X-ray micrographs of one non-infected *Acanthamoeba castellanii* (Fig. 3a) and Lurbovirus-infected amoebae with infection times ranging from 6 to 70 h (Fig. 3b–i). Here we identify several stages in the infection process. At 6–15 hpi (Fig. 3b–d) the cells have a vacuolated appearance, which is typical for a healthy amoeba of this kind (Fig. 3a). The small (< 500 nm) and close-to circular objects, probably mitochondria or storage granules, are distributed in all parts of the cytoplasm. In some images (e.g. Figs. 2b and 3c) the whole cell has adhered to the surface with extended pseudopods appearing as smooth gray areas around the edges of the cell.

The virions start to appear in the cells at 18 hpi (Fig. 3e) and the increasing number of particles is quite striking at long infection times of 54 h and 70 h (Fig. 3h–i). We also note that no virions are detected in the medium around the cells up to 18 hpi, indicating that observed virions are produced within cells rather than being residues from the infection process due to the high multiplicity of infection (MOI). At 18 hpi, free virions start appearing in increasing numbers, likely released from intact cells through exocytosis^16,20^ or originating from cells that have already ruptured and spilled their contents. Possible viral factories, similar to the one pointed out in Fig. 2b, can be seen in Fig. 3e–g as areas close to the nucleus with significantly lower concentration of X-ray absorbing objects.

The vacuolated structure of the cells disappears in a way that appears well correlated with the production of virions. Finally, we note that the nucleus, including the darker nucleolus, often stays intact up to the last stage of infection. This indicates that no virions are located in the nucleus. In the displayed images the nucleus can be seen up to 54 hpi (Fig. 3a–h). More X-ray micrographs at each considered infection time, along with a control series, are included in Supplementary Information.

### End-point dilution titrations and thermal gravimetric analysis (TGA)

A classical way to verify the average number of virions produced per cell, seen in the late stages of the infection, is to use end-point dilution titrations and calculate the average burst size. These experiments were done at 32 °C in medium containing 20 mM glucose, resulting in an infection process that proceeded much faster than in the X-ray imaging experiments, in line with published results for related viruses^11,18–20^. From direct counting of viral particles as well as limited dilution of the stock of purified Lurbovirus, we estimate that the non-infectious:infectious particle ratio is about 1:10. The end-point dilution titrations show that the output of new infectious particles per cell is between 280 and 660 (95% confidence interval).

**Figure 4:**
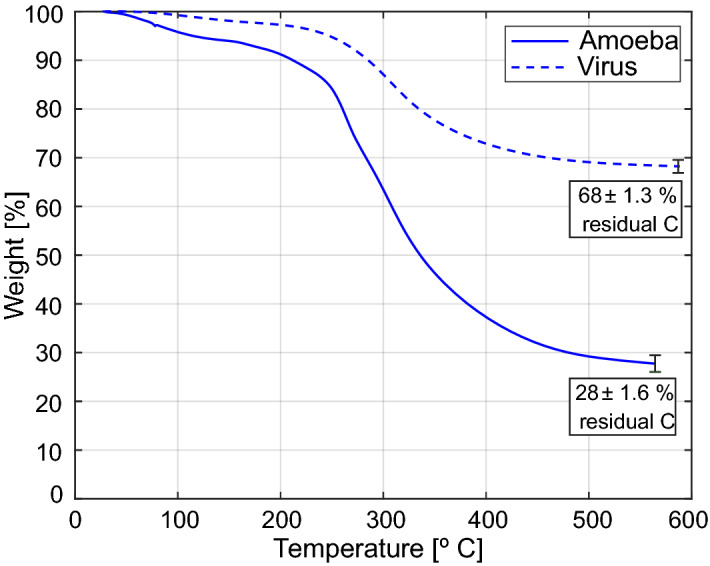
TGA thermograms of virus and cells. Solid line represents cells and dashed line represents virus. Measurements (n = 2) were performed under inert atmosphere. The residual mass after pyrolysis consists mostly of char, and was used to estimate the relative carbon content of virus (68%) and amoeba (28%). The error bars represent the standard error of the mean.

To gain further insight into the viral production mechanism the viral burst size can be used to estimate the biomass converted from cellular material to virus. Assuming the amoebae have an average volume of 3000 µm^3^^26^ and given that the virion in Fig. 1b has a volume of 0.23 µm^3^, the fraction of the host cell volume occupied by Lurbovirus at viral burst is estimated to be 2–5%. However, this ignores differences in density and biomass concentration between amoeba and virus, which is evident from the high contrast of the virions in the X-ray images. In order to determine this difference in biomass concentration, TGA was performed on both amoebae and virus. The results are shown in Fig. 4 and the measurement details are outlined in “Materials and methods” section. The residual weight, after pyrolysis, can be useful in predicting the relative amount of carbon content in these two samples, considering the similarity of biomolecules in these two organisms and their thermal decomposition behavior. The virus sample showed a weight reduction of 32% with a residual carbon content of 68%, while the amoebae lost about 72% of its weight, leaving behind about 28% carbon content. The residual carbon content may not be accurate, however, it is a good indicative for the prediction of the relative carbon content of virus and amoebae samples. These results show that the carbon content of the virus sample is about 2.5 times of that of amoebae. Similarly, the water content can be assumed to be 2.5 times lower in the virus sample compared to the amoebae. Taking this factor 2.5 into account for the difference in biomass concentration, a more accurate estimate of the biomass converted from cellular material to virus can be determined as 5–12%.

### X-ray image analysis of viral production and conversion of biomass

**Figure 5:**
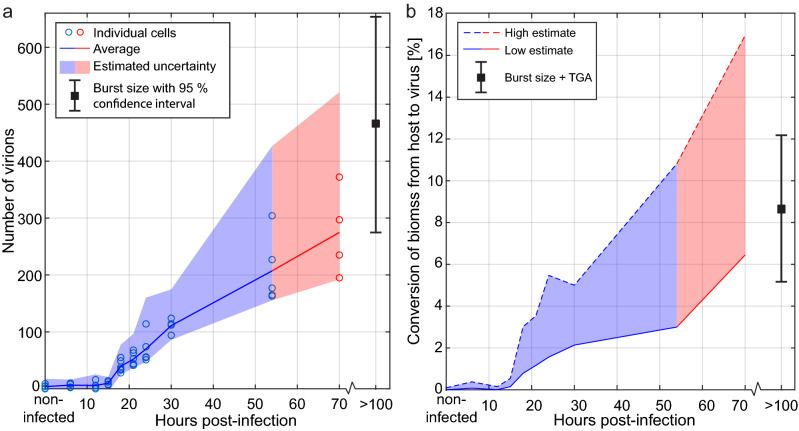
Virion production and conversion of biomass (C_1.3_ + N_1.9_ + O_0.1_) as function of infection time. (**a**) The number of virions were counted in 52 different cells with infection times ranging from non-infected (0 h) to 70 h. The data at 70 hpi should be seen as a more approximate count and is therefore displayed in red. Each blue or red ring corresponds to an individual amoeba, while the solid line shows the average at each infection time. An estimated uncertainty (“Materials and methods”) was calculated for each data point and is indicated by the shadowed area. In addition, the result of end-point dilution titrations (n = 15) is shown at > 100 hpi (black square), including a 95% confidence interval. (**b**) The conversion of biomass from host to virus is estimated by measuring the X-ray absorption in the virions compared to the whole cell, in 32 X-ray images with sufficient quality. A high estimate (dashed line) is calculated by summing the absorption in the areas in the images marked as virions. A more moderate estimate (solid line) is given by multiplying the average number of counted virions by the typical absorption in a single virion. The conversion given by the end-point dilution titrations, together with TGA, is shown at > 100 hpi. Note that end-point dilution titrations and TGA were performed at 32 °C in PPYG medium containing 20 mM glucose, whereas quantitative analysis of X-ray micrographs were performed at room temperature in PPYG medium containing no glucose.

A quantitative analysis of the X-ray microscopy data provides more information about the full viral replication cycle. Here we used 52 X-ray micrographs of cells infected for up to 70 h and non-infected reference cells. In the images acquired at 70 hpi the high virus concentration made it difficult to distinguish individual virions, requiring manual classification of virions. Thus, the numbers presented for this infection time should be considered as approximate counts and are therefore displayed in a different color in Fig. 5.

Figure 5a shows the number of identified virions inside the cells at each considered infection time. The data for each analyzed cell is plotted, together with the average number of virions at each infection time and an estimated uncertainty. Here, we also include the burst size at > 100 hpi, given by the end-point dilution titrations. It is clear from the results in Fig. 5a that the viral production takes off between 15 and 18 hpi. The production of new particles then continues throughout the time series without showing any signs of leveling out, reaching an average of almost 300 virions/cell at 70 hpi. Furthermore, we see that the quantitative analysis of the X-ray images agrees well with the results of the end-point dilution titrations.

The X-ray microscopy data also allowed for a more complete analysis of the conversion of biomass from host amoeba to virus, during the full viral replication cycle (Fig. 5b). This analysis was done by measuring the total X-ray absorption in the virions compared to the total X-ray absorption in each cell, resulting in an estimated conversion of biomass of 6–17% from amoeba to virus at 70 hpi. The high estimate was given by the total absorption in the area marked as virions, while the low estimate was given by the absorption of a typical virion multiplied by the number of identified virions. More details are provided in “Materials and methods”.

Altogether, Lurbovirus was found to infect *Acanthamoeba castellanii* at 32 °C in medium containing 20 mM glucose with a burst size of 280–660 virions/cell and conversion of biomass of 5–12% according to dilution titrations and TGA. At room temperature in medium without glucose, soft X-ray microscopy gave an average of almost 300 virions/cell at 70 hpi (Fig. 5a), corresponding to a conversion of biomass of 6–17% (Fig. 5b). At these conditions, the most striking morphological changes were the formation of a possible viral factory and the change in size and quantity of vacuoles during the infection (Figs. 2 and 3). On the contrary, the nucleus remained intact throughout the infection process. These morphological changes correlated well with those observed by TEM at 32 °C in medium containing 20 mM glucose (Fig. 1c and Supplementary Fig. S14).

## Discussion

In our X-ray measurements a negligible amount of virions is produced before 18 hpi and complete lysis occurs first at 54 hpi to 70 hpi. For *Pithovirus sibericum* complete lysis of infected cells occurs at approximately 15 hpi, which is preceded by the continuous release of virions via exocytosis, starting at 8 hpi^16^. Similarly, for Cedratvirus A11 mature virions are detected inside amoebae after 6–8 hpi, followed by partial burst starting at 10 hpi and complete lysis occurring at 24 hpi^18^. The prolonged infection process of Lurbovirus in this study compared to its relatives is attributed to that our amoeba cells are infected at room temperature in a medium without glucose and not inside an incubator at optimal conditions for cell replication. Control measurements in an incubator show that complete lysis occurs at approximately 24 hpi at 32 °C with 20 mM glucose in the medium, which supports this explanation. We note that our sample preparation procedure will bias us toward longer infection cycles, since the best X-ray micrographs will be obtained from adhered cells that tend to be less infected than cells that do not adhere. All things considered, this results in an infection process that is roughly three times slower than at optimal conditions and makes it possible to closely follow the progression of the viral replication cycle.

To gain further insight into the replication cycle, it is interesting to compare the morphological changes we observe with previous studies on giant DNA viruses using TEM. Our observation that the host nucleus and nucleolus appear unaffected throughout the replication cycle is consistent with previous observations for *Pithovirus sibericum*^11,16^ and *Cedratvirus getuliensis*^20^ and in contrast with the infection process for *Pandoraviridae*^11,14,15^. Their intracytoplasmic and nucleus-dependent replication modes, respectively, have been attributed to differences in gene content, virus-encoded functions and particle proteomes^11^. *Pandoraviridae* lack essential components of the DNA replication machinery^11^, such as a DNA ligase, topoisomerases and a DNA sliding clamp, whereas *Pithovirus sibericum*^3,18^ and Cedratvirus A11^18^ contain genes associated with DNA transcription and repair. This hints that Lurbovirus may also contain essential genes involved in replication of the viral genome, during which the host nucleus may not be actively involved. Keeping the nucleus intact, on the other hand, allows cellular functions to be sustained late through the replication cycle, which enables the large burst size. We observe the occurrence of a possible viral factory at room temperature from 18 hpi and onward, which is about three times slower than other *Pithoviridae* replicated at 32 °C and thus related to the infection conditions discussed above. For *Pithovirus sibericum* a viral factory is observed after 6–8 hpi^11,16^ and for Cedratvirus A11 it is present at 4 hpi^18^, in both cases just prior to the first observation of mature virions. We can only assign a viral factory simultaneously to the first observation of mature virions, which may be due to the vacuolated structure obscuring the assignment at earlier infection times. After that the number of vacuoles in the cell is quickly reduced, but their average size increases. This suggests that the viral infection results in a stress-related cellular response in the phagosome and contractile vacuole structure of the amoeba. The presence of clusters of virions inside vacuoles in e.g. Figure 3h indicates that many virions are released through exocytosis.

We find it necessary to account for difference in carbon density to accurately estimate conversion of biomass. The classical estimate from end-point dilution titrations combined with TGA is crude, because it ignores differences in individual cell volume^26^ and burst size^27^, for which the latter also depends on growth conditions^28,29^. Additionally, the sample preparation for TGA requires trypsinization of adherent cells and fairly low-viscous samples, which add an ambiguity to the initial conditions, i.e. to determine when weight stabilization has been achieved. We note that accurate quantitative conversion of biomass based on X-ray microscopy requires tomographic methods, since there is a large uncertainty in background normalization that mainly affects the estimated absorption of the host cell. Furthermore, since we cannot follow the cells to complete lysis at optimal growth conditions nor count virions released through exocytosis, we most likely underestimate the burst size of the Lurbovirus in our X-ray measurements. End-point dilution titrations at room temperature result in an estimated burst size of 30–330 virions/cell, meaning it is difficult to draw any definite conclusions regarding the effect of growth conditions on conversion rates. Nevertheless, our conversion estimate is supported by the two independent techniques and we declare the overlapping interval of 6–12% as our best guess of conversion of biomass for the Lurbovirus.

The impact of a viral infection on the host metabolism can be related to the host’s energy budget^29^. We estimate that our measured conversion of 6–12% corresponds to 11–21% of the host’s energy budget (Supplementary Information), which is a high consumption given the size of the amoebae. For comparison, trophic levels in ecosystems usually have a transfer efficiency of 10–13%^30^, whereas the influenza virus that infects mammalian cells of similar size only consumes ~ 1% of its host’s energy budget^35^.

Our estimate of burst size of 280–660 virions (95% confidence interval) is comparable to *Pithovirus sibericum*^16^, but slightly smaller than *Mimiviridae* and *Pandoraviridae*^11^. This would suggest that conversion of biomass from amoeba to virus may be higher for giant DNA viruses than an average eukaryotic viral infection. Although such a claim requires further experimental studies, in particular tomography on large cohorts of cells, it is tempting to speculate why this would be the case. Very high conversion above the host’s energy budget implies that storage granules and cell organelles are consumed during viral replication. This requires that viral proteins can retain core cellular functions and actively rewire the host metabolism to avoid encystment. The high degree of complexity in genomes of giant DNA viruses, encoding many putative proteins with unknown function, may therefore indicate that these viruses could hijack the cells and direct their function to suit the virus and its replication.

## Materials and methods

### Virus isolation and characterization

A soil sample was collected from the banks of a small stream southwest of Uppsala, Sweden. About 30 g of the soil sample was suspended in 100 ml PBS supplemented with 100 µg/ml of Amphotericin B. The mixture was incubated with gentle stirring for 24 h at 4 °C. After the incubation, the suspension was allowed to settle for 1 h to remove the large soil particulates. About 10 ml of the supernatant was pipetted and sequentially spun for 10 min at increasing relative centrifugal force (RCF) of 500*g*, 1000*g*, 4000*g*, 10,000*g* and 15000*g*. After each step of spinning the pellet was collected and the supernatant was subjected to the next higher RCF. Each of these pellets was resuspended in PBS containing 25 µg/ml amphotericin B, 50 µg/ml Ampicillin, 100 units Penicillin and 100 µg/ml of Streptomycin (all from Sigma Aldrich). The virus was eventually found in the 1000*g* pellet. *Acanthamoeba castellanii* (ATCC 30010) adapted to Amphotericin B and grown in PPYG medium^31^ in 24 well plates was used as bait for isolation of potential viruses. The same antibiotic and antifungal mixture with the same concentrations as above was also added to the medium.

The sample supernatant was added to the first well of each row of a 24 well plate at a ratio of 1:10 and serially diluted 1:10, 5 steps, one well in each row was kept as a control. The cells were inspected for signs of infection and the supernatant from selected wells were transferred to fresh cells in new 24-well plates and again serially diluted. One of several potentially infected samples was amplified in 75 cm^2^ culture flasks and the supernatant after cell lysis was purified on non-continuous sucrose gradients. The purified sample was placed on formvar/carbon-coated Cu grids (TedPella) and imaged (unstained) in a scanning transmission electron microscope (STEM) (Quanta 650 FEG) with an STEM II detector in high-angle annular dark-field mode (Thermo Fisher Scientific) using 30 kV acceleration voltage. The sample was also applied to a C-Flat grid CF-2/2-2C (Fischer Scientific), cryo-frozen and imaged in a 200 kV Talos Artica using a Falcon II detector (Thermo Fischer Scientific).

### Sample preparation for transmission electron microscopy

The cells were fixed in 2.5% glutaraldehyde in 0.1 M phosphate buffer at pH 7.4 and stored at + 4 °C until further processed. Following fixation, the cells were rinsed in 0.1 M phosphate buffer prior to post-fixation in 2% osmium tetroxide in 0.1 M phosphate buffer at pH 7.4 and 4 °C for 2 h. The cells were then stepwise dehydrated in ethanol followed by acetone and finally embedded in LX-112 (Ladd). Ultrathin sections (approximately 60–80 nm) were cut using a Leica EM UC 7 (Leica) and contrasted with uranyl acetate followed by lead citrate. The sections were imaged using the same STEM as in the previous section.

### Sample preparation for X-ray microscopy

Infection of *Acanthamoeba castellanii* with Lurbovirus was done in a two-step process, designed to produce a large fraction of simultaneously infected cells while still minimizing the number of excess virions in the medium. In the first step, *Acanthamoeba castellanii* (ATCC 30010) adapted to Amphotericin B (approx. ${10}^{6}$ cells/ml) in PPYG medium^31^ and Lurbovirus (approx. ${10}^{10}$ virions/ml) in PBS were added to 2 ml Eppendorf safe-lock tubes, with an MOI of 500. Both cell and virus media contained the same antibiotic and antifungal mixture with the same concentrations as above. The tubes were put on a slowly rotating stage to avoid sedimentation and left for 4 h to allow virus absorption. In the next step, the infected cells were seeded on Au TEM grids with a holey carbon layer (Agar Scientific) and placed in 4-well plates. After 2 h, the grids were carefully washed three times by removing the medium from the wells and adding new incomplete PPYG medium (without glucose) with a micropipette. The purpose of the wash was to dispose of non-absorbed virions around the cells and the incomplete growth medium was used to slow down cell metabolism and allow higher X-ray transmission due to its low carbon content. We estimate the X-ray transmission through 10 µm of incomplete growth medium to be 27%. After the wash was complete, the grids were left to let the infection proceed for a range of times, from 6 to 70 h, counting from when the virus was initially added to the cells.

All the steps above were performed in room temperature, giving the long infection times compared to previous studies on e.g.* Pithovirus sibericum*^16^ and Cedratviruses^18–20^. A control experiment was also done later, which shows that at 32 °C and with 20% glucose w.r.t. complete medium, the infection process is about a factor of two faster.

Before imaging, the grids were picked up and plunge-frozen in liquid ethane. By blotting the grids from behind and monitoring the process with a microscope, the liquid layer was kept slightly thinner than the grid height (10 µm). During the experiments the grids were kept at $- 165^\circ \mathrm{C}$ on a custom cryogenic sample holder.

### Laboratory full-field water-window cryo-microscopy

All X-ray imaging was performed using the Stockholm laboratory X-ray microscope, previously described in Refs.^32,33^, which operates in the water window. Recent improvements to the microscope have resulted in increased X-ray flux and more stable and reliable operation^32^, thus allowing longer and more complex studies of biological samples. The arrangement is based on the $\lambda =2.48$ nm (500 eV) hydrogen-like (NVII) line-emission from a liquid-nitrogen-jet laser plasma. The plasma is generated by focusing a $\lambda =1064$ nm beam of a 2 kHz, 600 ps diode-pumped Nd:YAG slab laser (Fraunhofer ILT, Aachen)^34^ at a typical average power of 40–60 W onto a 30 µm diameter liquid nitrogen jet. Improvements to the liquid jet stability have recently been implemented^33^, which have increased the over-all stability and reliability of the setup. The plasma can be kept at a large distance (typically > 3.5 mm) from the nozzle orifice, which is necessary to maintain stability during long-term operation.

A 58 mm diameter Cr/V multilayer condenser mirror (radius of curvature 350 mm) (Optix fab, Jena) is used to image the 30 µm jet with $1.6\times$ magnification onto the sample. With its 500 double-layer, $\Delta \lambda /\lambda =450$ bandwidth and $R=4.66$ average reflectance at $\lambda =2.48$ nm, the mirror also acts as a monochromator. A circular central stop is placed before the sample to create a hollow-cone illumination and a 200 nm Al filter blocks stray light from the plasma and the laser. The sample grids are mounted on a custom cryogenic sample holder (Gatan), which is then inserted into a modified TEM goniometer stage (FEI). The stage allows fine movement in all directions as well as rotation for tomography.

The Ni zone plate objective has a 30 nm outermost zone width and a 200 µm diameter, giving a quite long working distance of $f=2.42\,\mathrm{ nm}$ at $\lambda =2.48\,\mathrm{ nm}$. Even though the number of zones ($N=1667$) is larger than the estimated source bandwidth ($\Delta \lambda /\lambda =700-1000)$^35^, chromatic aberrations are not limiting the resolution in the current setup. A cooled back-illuminated CCD (iKon-L, Andor) with 2048 × 2048 13.5 µm pixels is finally used to record the images. A magnification of $M=700$ was used in all X-ray images, giving a pixel size corresponding to 19.3 nm in the sample plane and a field of view (FOV) of 39.5 µm. This is large enough to fit the often quite large amoebae in the FOV, while not limiting the resolution.

### Data acquisition

A large number of samples (~ 40 grids) was imaged in order to collect data for the infection times ranging from 6 to 70 hpi, as well as non-infected cells and separate viral particles for reference. Each sample grid was scanned through using 1-s exposures to find specimen of interest in areas with a suitable ice-layer thickness. The final images were recorded using 60-s exposures (or by adding together two 30-s exposures). The slightly lower laser power (40–60 W) and thus longer exposure times than presented in Fogelqvist et al*.*^32^ was chosen to avoid putting too much strain on the microscope and thereby allowing for extensive long-term experiments. The photon flux at the detector during a 60-s exposure is approximately 700 photons per pixel without sample (i.e. flat-field recordings) and 130 photons per pixel with a typical cryo-fixed sample. The dose delivered to the sample at such an exposure is estimated to ${10}^{6}$ Gy.

### Image analysis and data processing

All images were smoothed by applying a Gaussian filter (${e}^{-\frac{{r}^{2}}{2{\sigma }^{2}}}$), where $\sigma =1$ pixel. Contrast and brightness have been adjusted for optimal viewing, whereas quantitative analysis was performed on absolute image channel values related to photon counts. For the quantitative analysis of viral growth 52 X-ray images of good quality were selected out of 214 imaged cells. These included 4–6 images acquired at each of the considered infection times (6 hpi, 12 hpi, 15 hpi, 18 hpi, 21 hpi, 24 hpi, 30 hpi, 54 hpi, 70 hpi) as well as non-infected cells acquired 24 h after transfer to incomplete PPYG medium (no glucose) for reference. X-ray micrographs of control cells acquired 4 h, 48 h and 72 h after transfer to incomplete PPYG medium are also presented in Supplementary Information. All high-contrast objects inside the cells were manually marked with a cross indicating their length and width (Supplementary Information Section C1). The marked images were then automatically analyzed and counted in MATLAB (R2018a) using a custom-made script. To categorize the marked objects, i.e. to separate virions from other objects, a minimum length (700 nm), maximum length (1500 nm) and minimum ellipticity ($\mathrm{length}/\mathrm{width}=1.3$) were set. All marked objects were visually inspected after automatic classification to verify the accuracy of the manual marking. The segmentation criteria were verified on known virions imaged outside of cells. Note that the other high-contrast objects in the cells consist mainly of close-to circular objects < 500 nm in size, believed to be mitochondria or storage granules^24^.

The images acquired at 70 hpi were generally more difficult to analyze due to the high concentration of overlapping virions. Therefore, an estimate of the number of virions was obtained by manually marking all particles resembling the virions identified in the samples up to 54 hpi. Thus, the data from 70 hpi cells are displayed in a separate color in Fig. 5.

An error analysis was done by first considering the objects that were (incorrectly) characterized as virions in the non-infected cells. By scaling the average number of incorrectly characterized virions with the total number of marked objects, an error was estimated for each data point. In addition, assuming that the 1-µm long virions were randomly oriented in space, about 30% would have a projected length < 700 nm and thereby not be counted. These two error sources were added to give the estimated total error shown as a shadowed area around the data points in Fig. 5a.

The conversion of biomass from amoeba to virus was estimated by considering the X-ray absorption in the virions compared to the whole cell. For this analysis the images needed to be normalized by the illumination background. Since the illumination is not known (and varying from image to image) it was estimated for each individual image by fitting a 2D Gaussian function to sampled points around the cell. To compensate for absorption in the medium, the estimated illumination was increased by a factor corresponding to absorption in a 4 µm ice layer (i.e. estimated typical thickness of a cell). 32 X-ray images with good quality and a sufficiently large open area around the cell were deemed suitable for this analysis. The absorption was finally calculated by normalizing by the estimated illumination, taking the negative logarithm (i.e. inverted form of Beer-Lambert law) and then summing the pixel values. Based on that amoebae have approximately 2/3 water content^36,37^, about 15% of the X-ray absorption in the cell is due to water and the calculated absorption (that is indicative of biomass) was decreased correspondingly. Assuming the water content is proportional to the weight loss measured by TGA (cf. Fig. 4), the corresponding value for the virions is about 3% X-ray absorption due to water.

The total absorption within each cell was compared to the absorption in the areas marked as virions (high estimate) or to the absorption in a typical virion multiplied by the number of identified virions (low estimate). The mean values for each infection time is shown in Fig. 5b. The typical absorption in a virion was measured in X-ray images containing only the virus. We note that the high estimate will also include the absorption of the cell volume above and below the identified virions, due to the nature of projection imaging. On the other hand, both estimates will fail to count all virions that could not be identified, as well as virions that have left the cell through exocytosis, which is especially important at longer infection times where the viral concentration is high.

### End-point dilution titrations

A viral stock of Lurbovirus was generated by infecting ten 175 cm^2^ flasks (Sarstedt) containing tropozoites of *Acanthamoeba castellanii* (ATTC 30010) in PPYG medium at a confluency of about 70–80% with Lurbovirus with an MOI of 10. The newly generated virus was purified using standard techniques^31^ and the quality of the preparation was verified by direct observation using STEM (Quanta 650 FEG with a STEM detector, FEI). The titer of the total viral stock was determined by standard limited dilution titration, starting with 0.01 ml and using tenfold dilutions. Infection was detected at a dilution of ${10}^{-8}$, but never at ${10}^{-9}$, and a few repetitions produced the same results. Therefore, the titer was estimated to be on the order of ${10}^{-10} {\mathrm{ml}}^{-1}$. We note that a rough estimate of the titer was sufficient at this point, while the latter experiments required a more accurate analysis. The total number of particles was also determined by direct counting in a manual cell counter (Improved Neubauer, chamber depth 0.01 mm, Marienfeld) using standard techniques.

For each of the experiments to determine the number of viruses generated per cell, 2 × 10^6^ tropozoites were seeded and allowed to adhere in 100 mm diameter tissue culture dishes (Sarstedt). The PPYG media in this case contained a reduced glucose concentration, 20 mM instead of 100 mM, to better investigate the true cell to virus conversion. 10^7^ of infectious Lurbovirus particles (MOI of 5) were then added to the cells in a reduced volume of PPYG medium (total of 3 ml) and incubated for 1 h, after which an additional 7 ml of medium was added. The infection process proceeded until almost all cells were completely lysed and only smaller pieces of cell debris remained (4–5 days).

The growth surface was scraped with a cell scraper to suspend as much as possible of the virus and remains of cells from the surface and the suspension was aspirated. This experiment was repeated 4 times with the titrations below repeated 4 times for each experiment, except for one of the titrations that failed, giving a total of 15 complete titrations. This virus suspension was then titrated with tenfold dilutions in a 24-well plate to get a rough estimate of the virus titer. In almost all the cases this titration yielded a dilution of 10^–9^. To make a finer titration, the same tenfold titration was made but this time the 10^–6^ well was further titrated 1:2 until the last well in the same plate, except for the two last wells that were left as uninfected controls. The 10^–6^ well, is well 0 in this titration series. Out of the 15 complete titrations, virus was detected in 15/15 wells at a dilution of ${2}^{-9}$, 12/15 wells at ${2}^{-10}$, 2/15 wells at ${2}^{-11}$ and then no further detection. This can be related to the fifty-percent tissue culture infective dose (TCID_50_), which should be found at a dilution between ${2}^{-10}$ and ${2}^{-11}$.

A more accurate estimate of the number of infectious virions produced per cell is given by statistical analysis. At each titration step the number of virions, $k$, in the different wells is assumed to be Poisson distributed, $P(k|\lambda )$, where $\lambda$ is the expected value. The chance of detecting virus in a well should thus be $1-P\left(0|\lambda \right)$, assuming that all virions are infectious. The expected number of virions per well, ${\lambda }_{i}$, at step $i$ depends on the unknown starting value$, a,$ and the known dilution factor$, {d}_{i}$, according to ${\lambda }_{i}=a{d}_{i}$. Thus, the starting number of virions (at titration step 0) can be obtained by fitting the resulting percentage of virus detection at each titration step, $i$, to the expected function, $1-P\left(0|a{d}_{i}\right)$. The error bar displayed in Fig. 5a is the 95% confidence interval of this fit.

The end-point dilution titrations presented above were performed at 32 °C, resulting in complete lysis at about 24 hpi. To evaluate the effect of temperature on burst size, tenfold dilutions were also performed at room temperature. Out of 17 complete titrations, virus was detected in 17/17 wells at a dilution of 10^–4^, 16/17 wells at 10^–5^, 5/17 wells at 10^–6^ and then no further detection. This means that TCID_50_ should be found at a dilution between 10^–5^ and 10^–6^, which corresponds to 30–330 virions/cell.

### Thermal gravimetric analysis (TGA)

*Acanthamoeba castellanii* trophozoites were harvested and washed with PBS two times by repeated centrifugation at 600*g* for 5 min and suspension. Before TGA, the cells were pelleted by centrifugation at 1200*g* for 10 min to be able to remove as much PBS as possible but still keeping the cells intact. Lurbovirus from a gradient-purified virus stock was prepared in a similar manner as the amoebae, except that the centrifugations during the washing steps to replace the storage buffer with PBS were performed at 4000*g* for 15 min and 6000*g* for 20 min during the pelleting step.

TGA measurements on amoeba and virus samples were performed using TGA 55 system (TA Instruments) under inert (N_2_) atmosphere using a heating rate of $2^\circ \mathrm{C}/\mathrm{min}$. During the pyrolysis it is expected that volatile compounds, water vapor and some degradation products, will leave the medium, while the large organic molecules will break down into smaller molecules and the organic content will mostly convert to char (C), remaining as solid inorganic residue. Evolved gas analysis was performed for the identification of pyrolysis products using a TGA-IR coupling system (Thermo-Fisher TGA-IR coupling system), connected to a Fourier-transform IR spectroscope (FTIR, Thermo Scientific Nicolet iS10) to obtain IR spectra in the range from 4000 to 450 cm^−1^. Traces of NO_2_ were observed either as a degradation product or a result of nitrogen gas reacting with the oxygen in the organic matter. Water traces were observed, in agreement with the weight loss of the sample. Most of the water in the samples was structural water, which required a higher energy barrier to be released from the sample. As low heating rate was used, the process was sustained and the weight stabilization was achieved at slightly higher temperatures.

## Supplementary information

Supplementary Information 1.

Supplementary Information 2.

## Data Availability

All data needed to evaluate the conclusions in the paper are present in the paper and Supplementary information. Additional data related to this study are available upon request from corresponding authors.

## Code availability

The code used to process the data that support the findings in this study are available in Supplementary information.
