# Supplementary material for: Quantitative conversion of biomass in giant DNA virus infection
Source: Sci Rep. 2021 Mar 3;11:5025. doi: 10.1038/s41598-021-83547-9 (PMC7930090; doi:10.1038/s41598-021-83547-9)
Supplement: Supplementary file 2 — Supplementary Information 2. [file 41598_2021_83547_MOESM2_ESM.pdf]

Supplementary Information

Quantitative conversion of biomass in giant DNA virus infection

Mikael Kördel[1,*], Martin Svenda[1,2], Hemanth K. N. Reddy[2], Emelie Fogelqvist[1], Komang G. Y. Arsana[1], Bejan Hamawandi[1], Muhammet S. Toprak[1], Hans M. Hertz[1], Jonas A. Sellberg[1,*]

* Corresponding authors (e-mail: jonassel@kth.se, mikael.kordel@biox.kth.se)

Affiliations
[1] *Biomedical and X-ray Physics, Department of Applied Physics, AlbaNova University Center, KTH Royal Institute of Technology, SE-106 91 Stockholm, Sweden.*

[2] *Laboratory of Molecular Biophysics, Department of Cell and Molecular Biology, Uppsala University, Husargatan 3 (Box 596), SE-751 24 Uppsala, Sweden*

CONTENTS

A. Supplementary texts **2**

A.1. X-ray microscopy of virus infections *2*

A.2. Relationship between conversion of biomass and energy consumption *2*

B. Supplementary figures **4**

C. Supplementary methods **13**

C.1. Marked example images for viral particle identification *13*

C.2. MATLAB scripts to identify viral particles in marked images *16*

C.3. MATLAB scripts to calculate the x-ray absorption in viral particles and cells *17*

D. Supplementary tables **18**

E. References **20**

A. Supplementary texts

A.1. X-ray microscopy of virus infections

We present all additional x-ray micrographs of non-infected (Fig. S1-S4) and virus-infected (Fig. S5-S13) amoebae that were used to study the viral replication cycle. The infection times range from 6 h (Fig. S5) up to 70 h (Fig. S13). All images were acquired using the Stockholm laboratory x-ray microscope with exposure times of mainly 60 s. For comparison, transmission electron microscopy of sectioned, stained, resin-embedded, amoeba cells are shown for non-infected (Fig. S14a) and virus-infected (Fig. S14b-c) amobae.

A.2. Relationship between conversion of biomass and energy consumption

Our experimental measure of conversion of biomass based on the high carbon-to-water contrast present in the water window can be related to the energy consumption needed to produce the virions inside the host cell. Following the same approach as Mahmoudabadia *et al.* (*1*), which is based on the equations presented by Lynch and Marinov (*2*), we assume that the basal metabolic requirement of a cell scales with cell volume as

$$E_M \approx 0.39V^{0.88}\ [10^9\ \mathrm{ATP/cell/h}], \tag{S1}$$

whereas metabolic requirement for cellular growth scales similarly as

$$E_G \approx 27V^{0.97}\ [10^9\ \mathrm{ATP/cell}]. \tag{S2}$$

This results in that the total energy budget of a cell during the cell division time *t* [h] is

$$E_C = E_G + tE_M. \tag{S3}$$

Since the cell metabolism is slowed due to the incomplete medium without glucose, we estimate the cell division time to be approximately the same as the last infection time, *i.e.* $t = 70$ h. The virus consumption of the host's energy budget is thus E_V/E_C, given that the energetic cost of the

virus E_V can be estimated. On the other hand, the cell also has energy stored in its cytoplasm and organelles, which must also correspond to E_G so that the total energy bound in the cell is

$$E_T = 2E_G + tE_M. \tag{S4}$$

Assuming that our x-ray contrast is proportional to the number of glucose molecules supplied to build the cell, which in turn is proportional to E_T, we can write conversion of biomass from cell to virus as

$$X_{CV} = E_V/E_T. \tag{S5}$$

Thus, the virus consumption of the host's energy budget becomes

$$\frac{E_V}{E_C} = \frac{X_{CV}E_T}{E_C} = X_{CV}\left(\frac{2E_G+tE_M}{E_G+tE_M}\right). \tag{S6}$$

Eq. (S6) relates conversion of biomass to virus consumption of the host's energy budget as defined in Mahmoudabadia *et al.* (*1*). Given that the amoebae have an average volume of 3000 μm^3 (*3*) and the cell division time is assumed to be 70 h, the virus consumption of the host's energy budget will be 67% larger than the measured conversion of biomass.

B. Supplementary figures

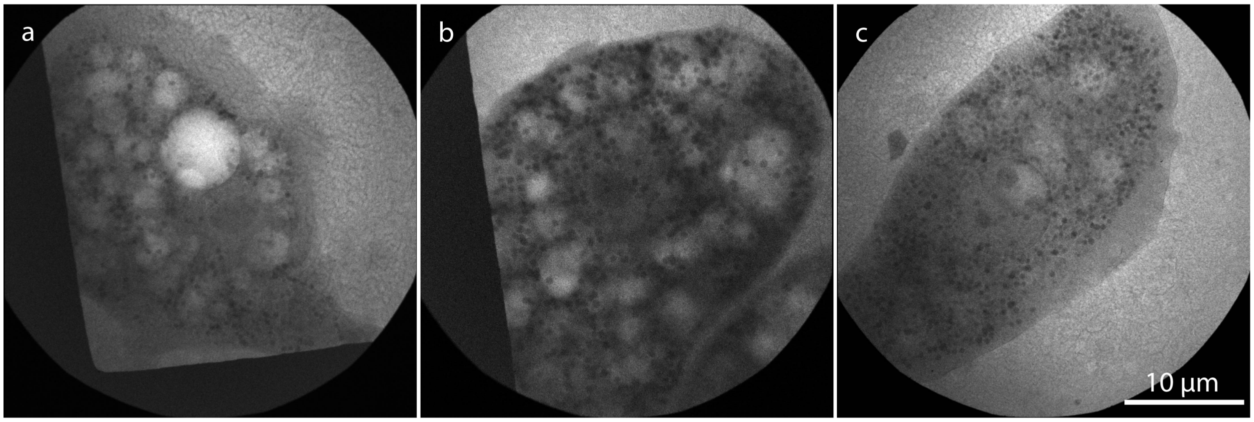

Supplementary Figure S1. Laboratory cryogenic x-ray microscopy of healthy, non-infected amoebae imaged 4 h after transfer to incomplete PPYG medium (without glucose). Scale bar is 10 µm and is valid for all images.

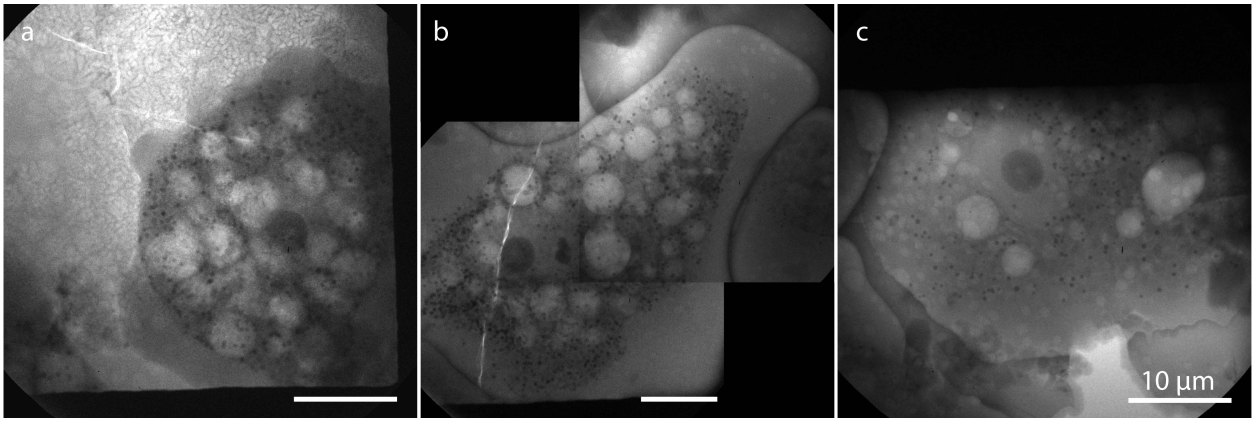

Supplementary Figure S2. Laboratory cryogenic x-ray microscopy of healthy, non-infected amoebae imaged 24 h after transfer to incomplete PPYG medium (without glucose). Note that the center image **(b)** consists of two images that have been stitched together. All scale bars are 10 µm.

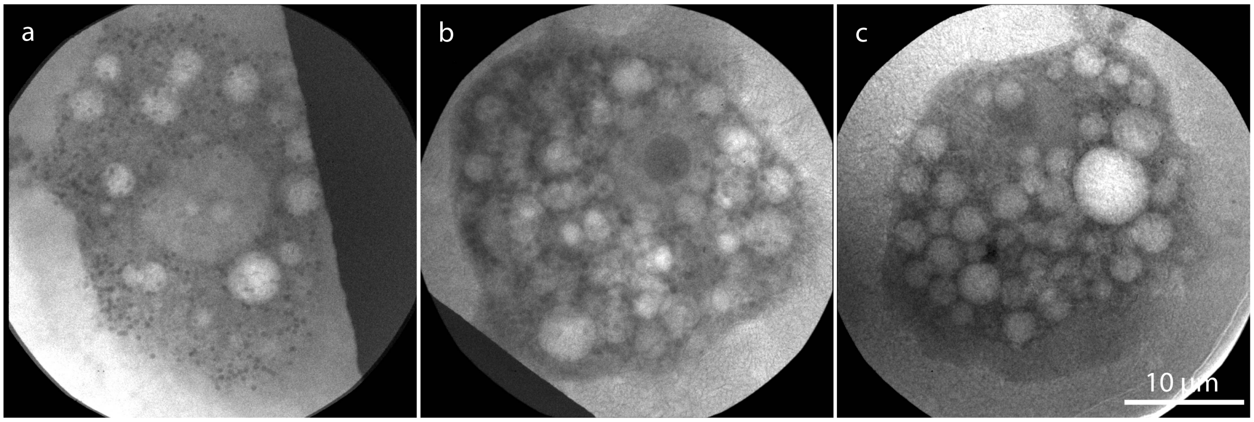

Supplementary Figure S3. Laboratory cryogenic x-ray microscopy of healthy, non-infected amoebae imaged 48 h after transfer to incomplete PPYG medium (without glucose). Scale bar is 10 µm and is valid for all images.

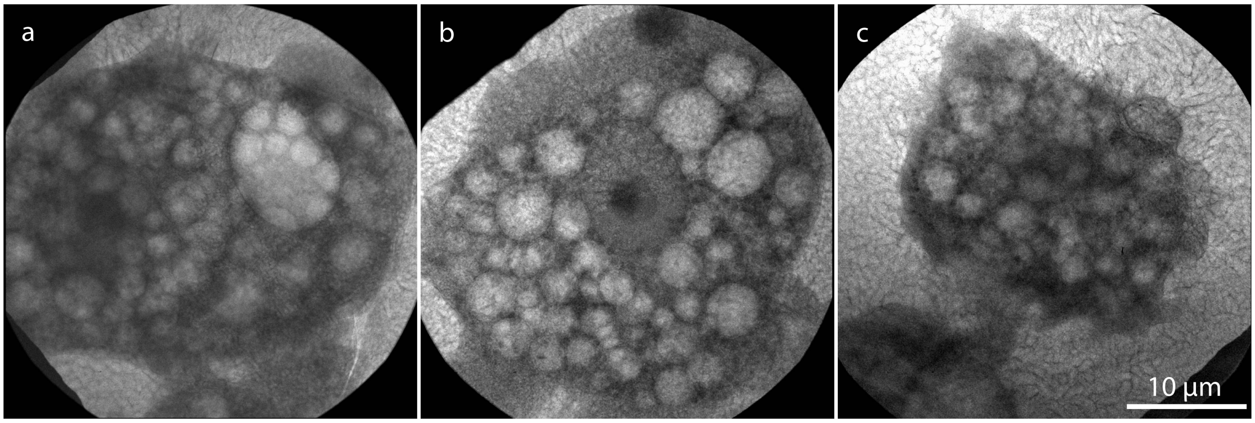

Supplementary Figure S4. Laboratory cryogenic x-ray microscopy of healthy, non-infected amoebae imaged 72 h after transfer to incomplete PPYG medium (without glucose). Scale bar is 10 µm and is valid for all images.

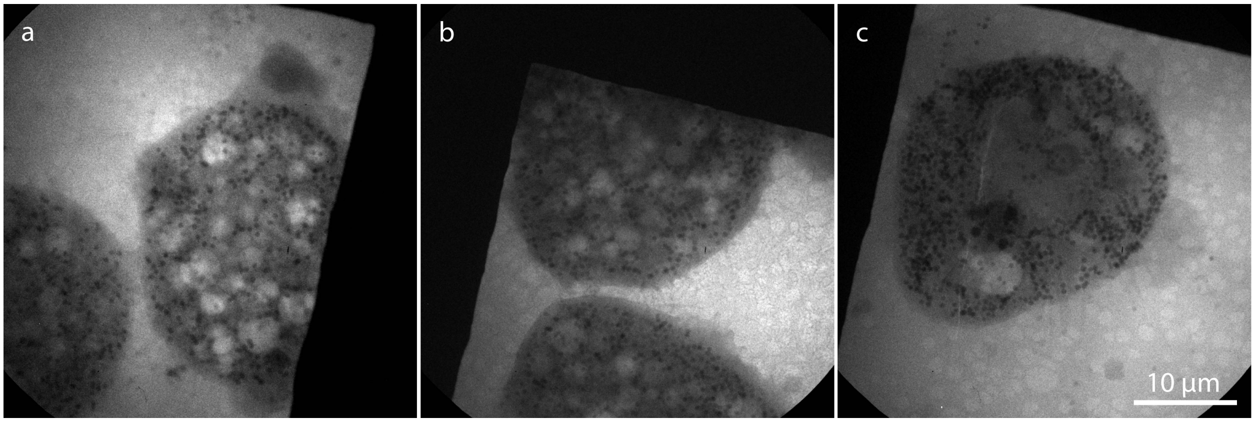

Supplementary Figure S5. Laboratory cryogenic x-ray microscopy of virus-infected amoebae imaged 6 hpi. Scale bar is 10 µm and is valid for all images.

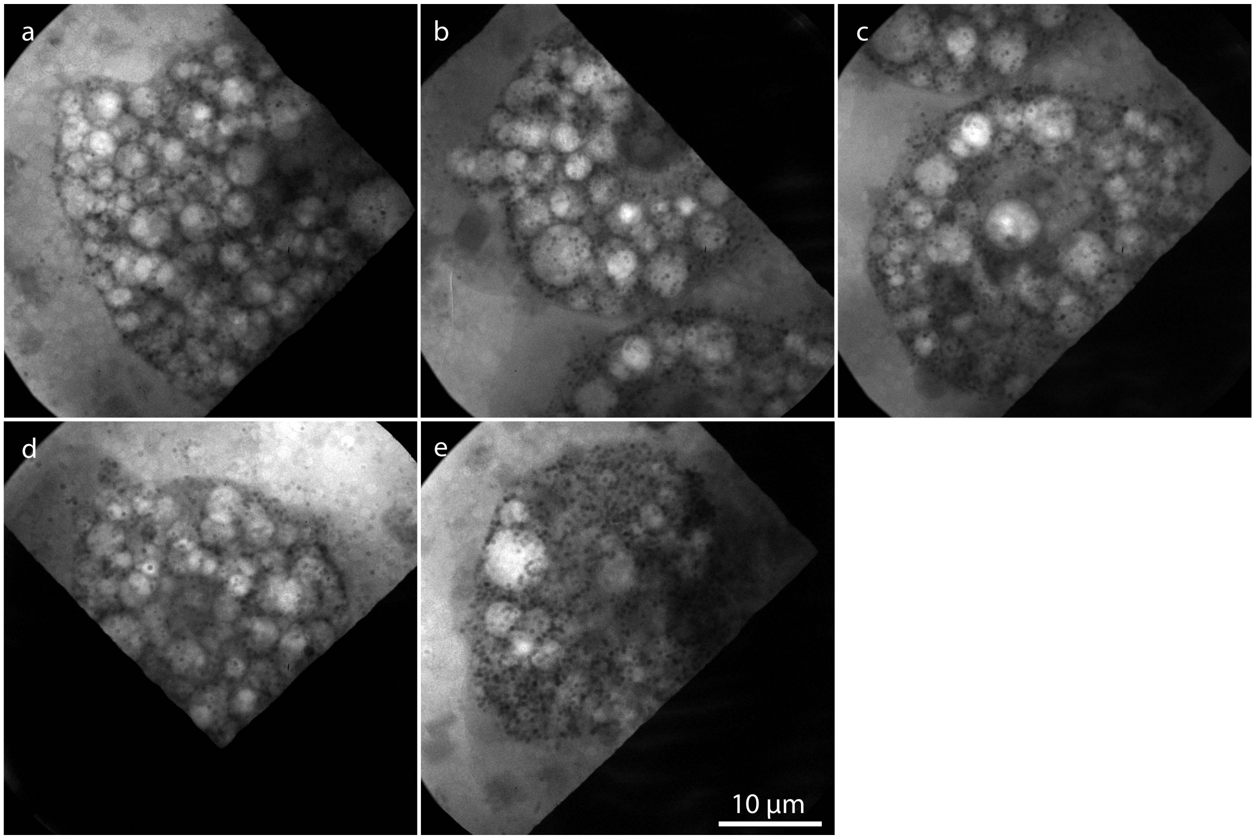

Supplementary Figure S6. Laboratory cryogenic x-ray microscopy of virus-infected amoebae imaged 12 hpi. Scale bar is 10 µm and is valid for all images.

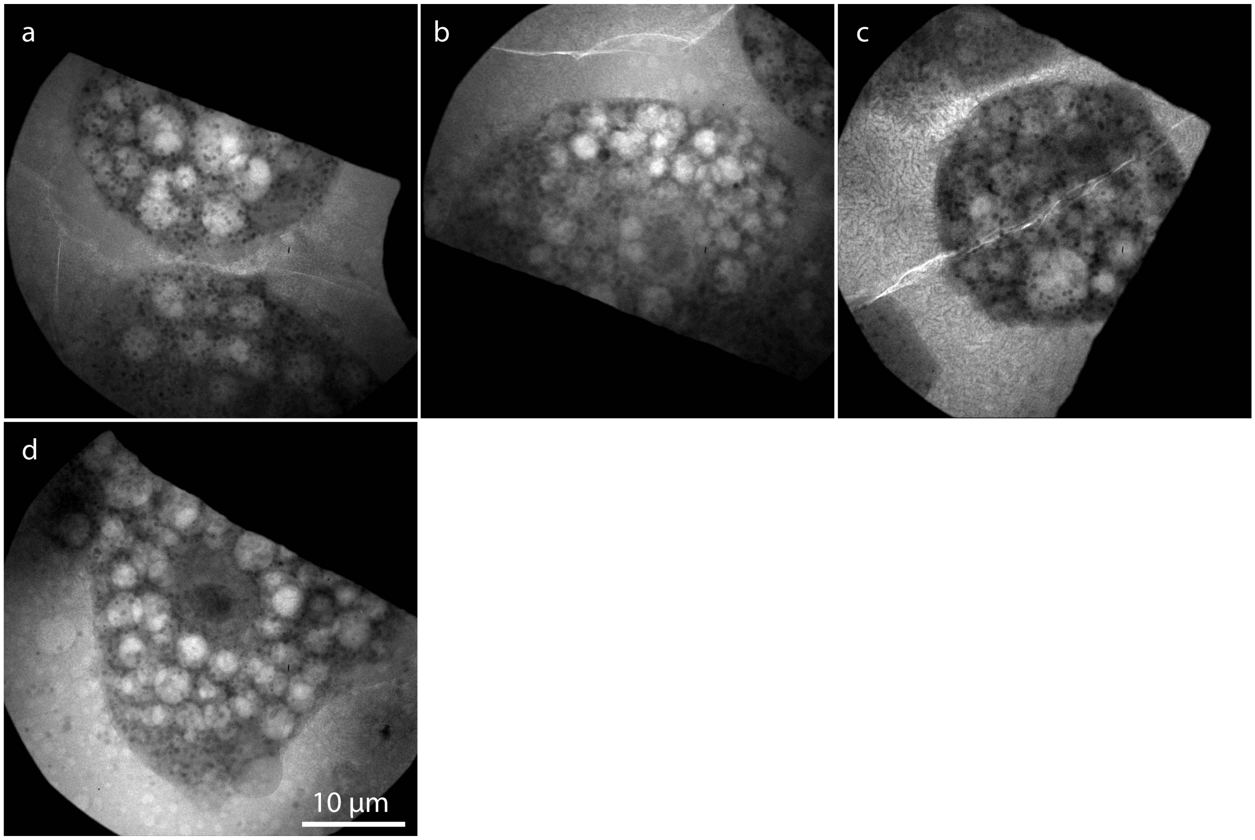

Supplementary Figure S7. Laboratory cryogenic x-ray microscopy of virus-infected amoebae imaged 15 hpi. Scale bar is 10 µm and is valid for all images.

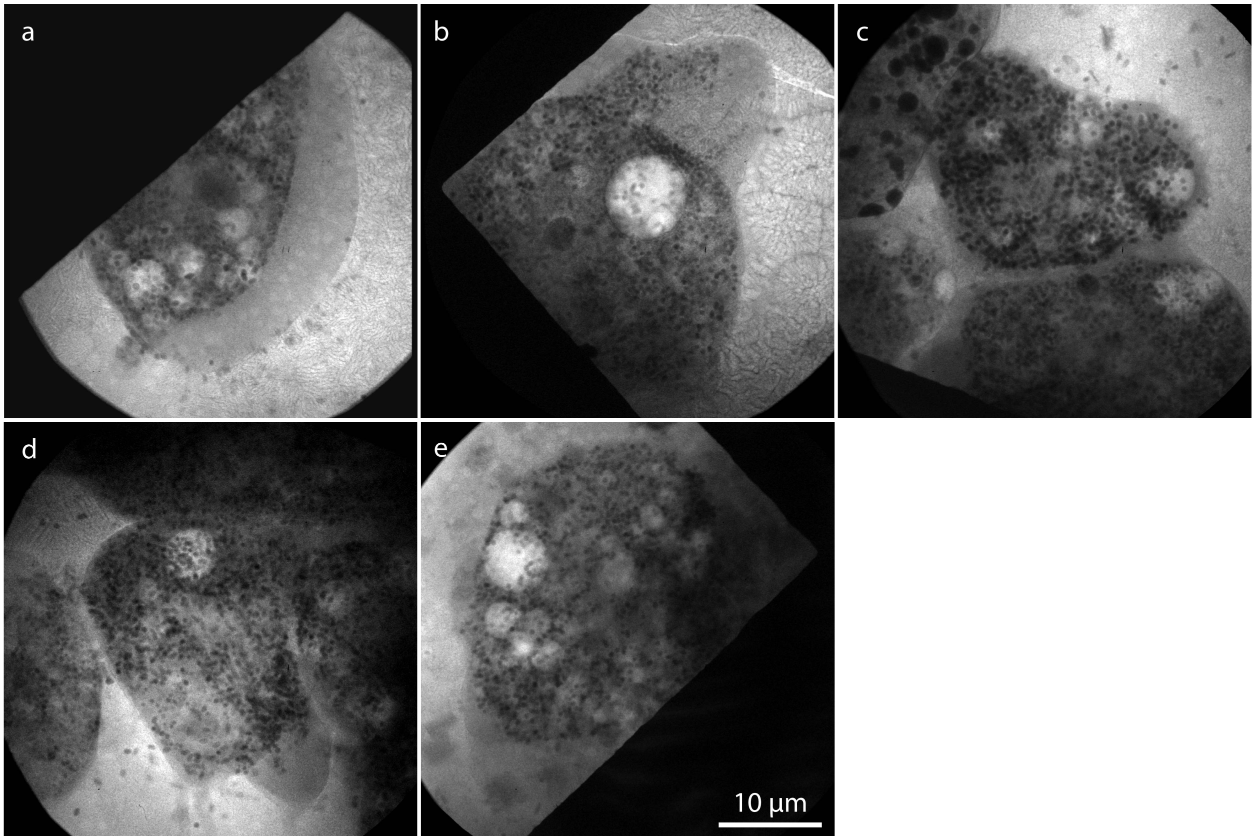

Supplementary Figure S8. Laboratory cryogenic x-ray microscopy of virus-infected amoebae imaged 18 hpi. Scale bar is 10 µm and is valid for all images.

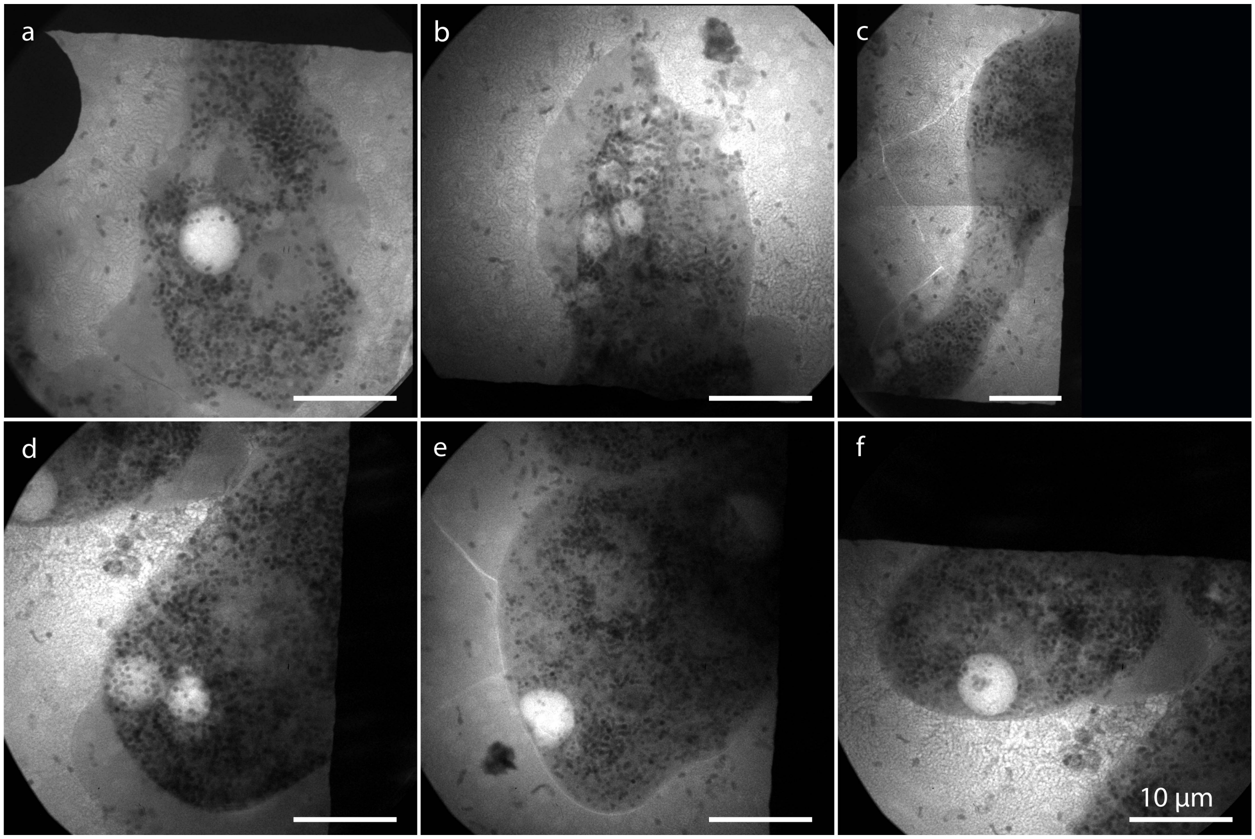

Supplementary Figure S9. Laboratory cryogenic x-ray microscopy of virus-infected amoebae imaged 21 hpi. Note that the top right image **c** consists of two images that have been stitched together. All scale bars are 10 µm.

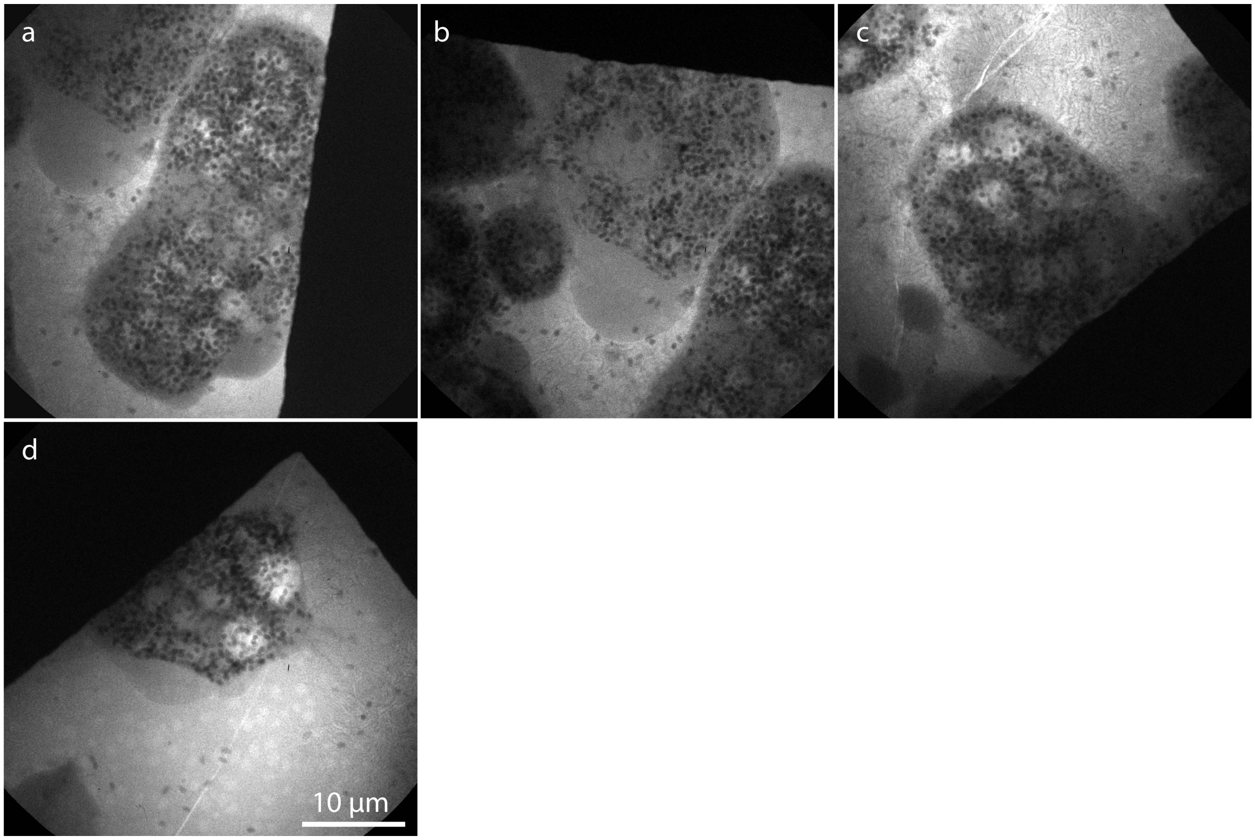

Supplementary Figure S10. Laboratory cryogenic x-ray microscopy of virus-infected amoebae imaged 24 hpi. Scale bar is 10 µm and is valid for all images.

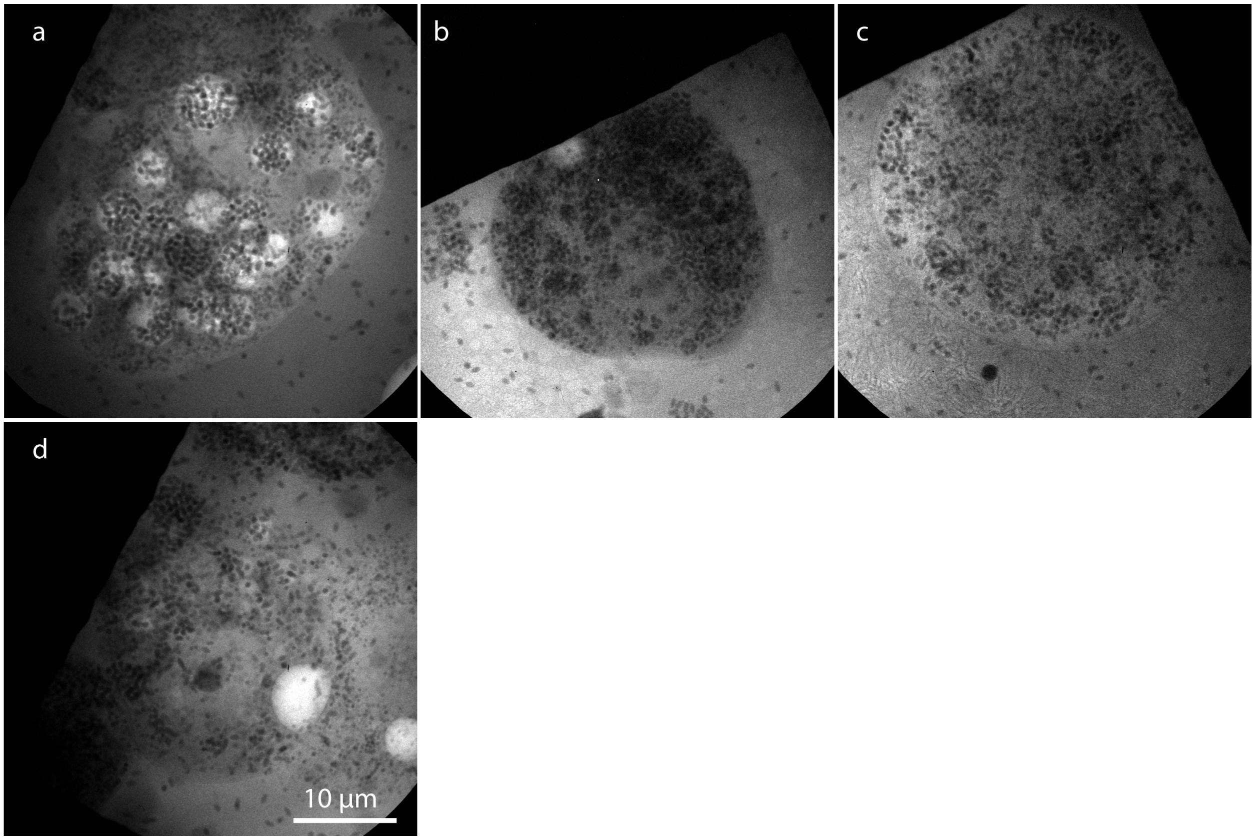

Supplementary Figure S11. Laboratory cryogenic x-ray microscopy of virus-infected amoebae imaged 30 hpi. Scale bar is 10 µm and is valid for all images.

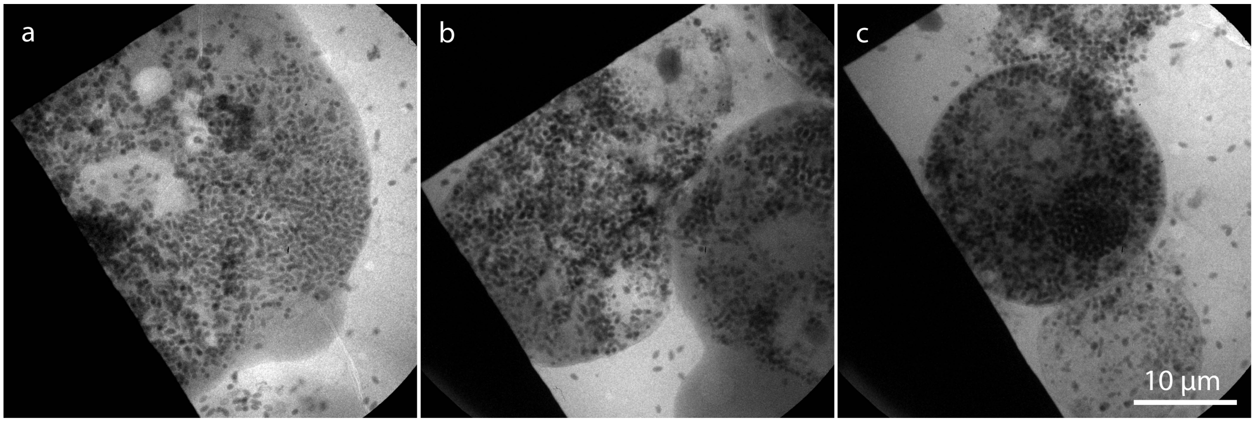

Supplementary Figure S12. Laboratory cryogenic x-ray microscopy of virus-infected amoebae imaged 54 hpi. Scale bar is 10 µm and is valid for all images.

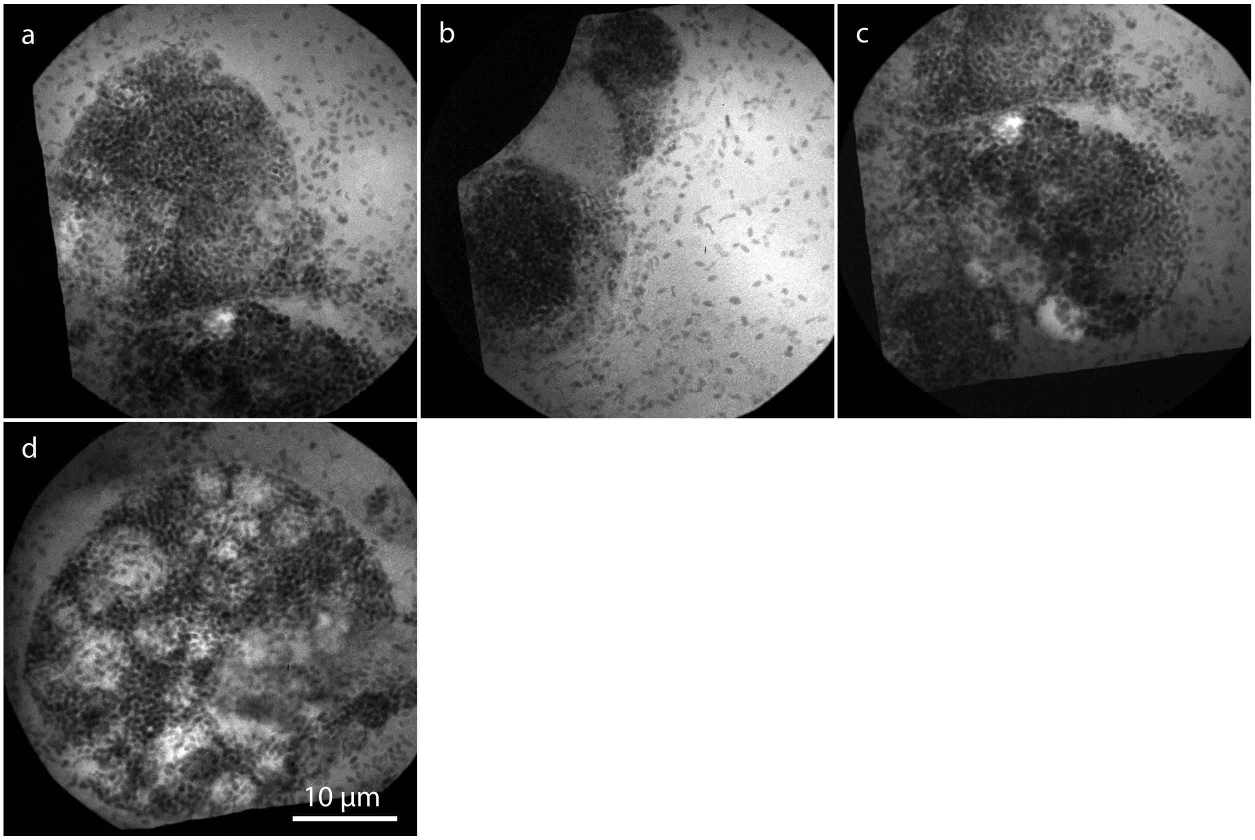

Supplementary Figure S13. Laboratory cryogenic x-ray microscopy of virus-infected amoebae imaged 70 hpi. Scale bar is 10 µm and is valid for all images.

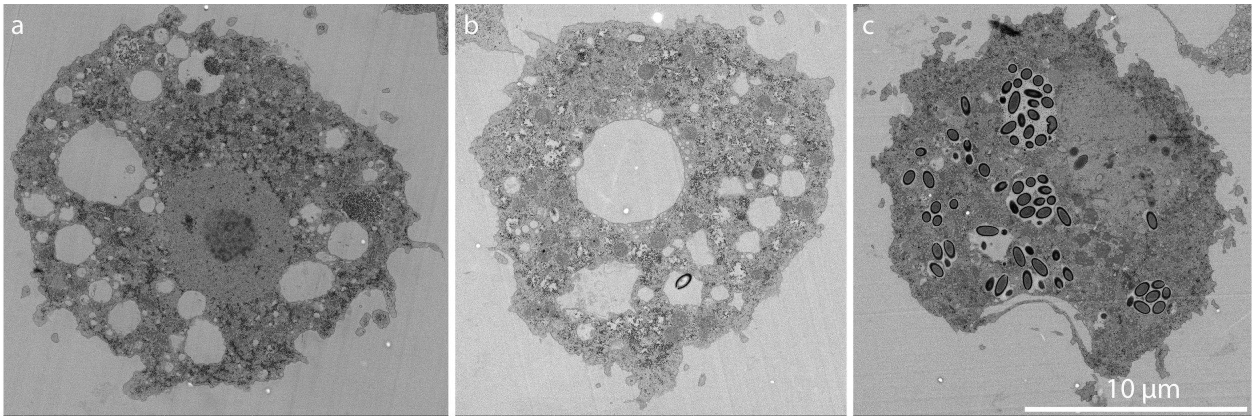

Supplementary Figure S14. Transmission electron microscopy of sectioned, stained, resin-embedded, amoeba cell (at 32ºC in medium containing 20 mM glucose) imaged (**a**) prior to infection (healthy amoeba), (**b**) 2 hpi (early stage of infection) and (**c**) 18 hpi (late stage of infection). Scale bar is 10 µm and is valid for all images.

C. Supplementary methods

The source code used to analyze the raw data presented in the main article is comprised of 7 MATLAB (R2018a) scripts available in a separate zip-file and whose input and docstrings are presented below.

C.1. Marked example images for viral particle identification

Three example images used as input for viral particle identification using MATLAB.

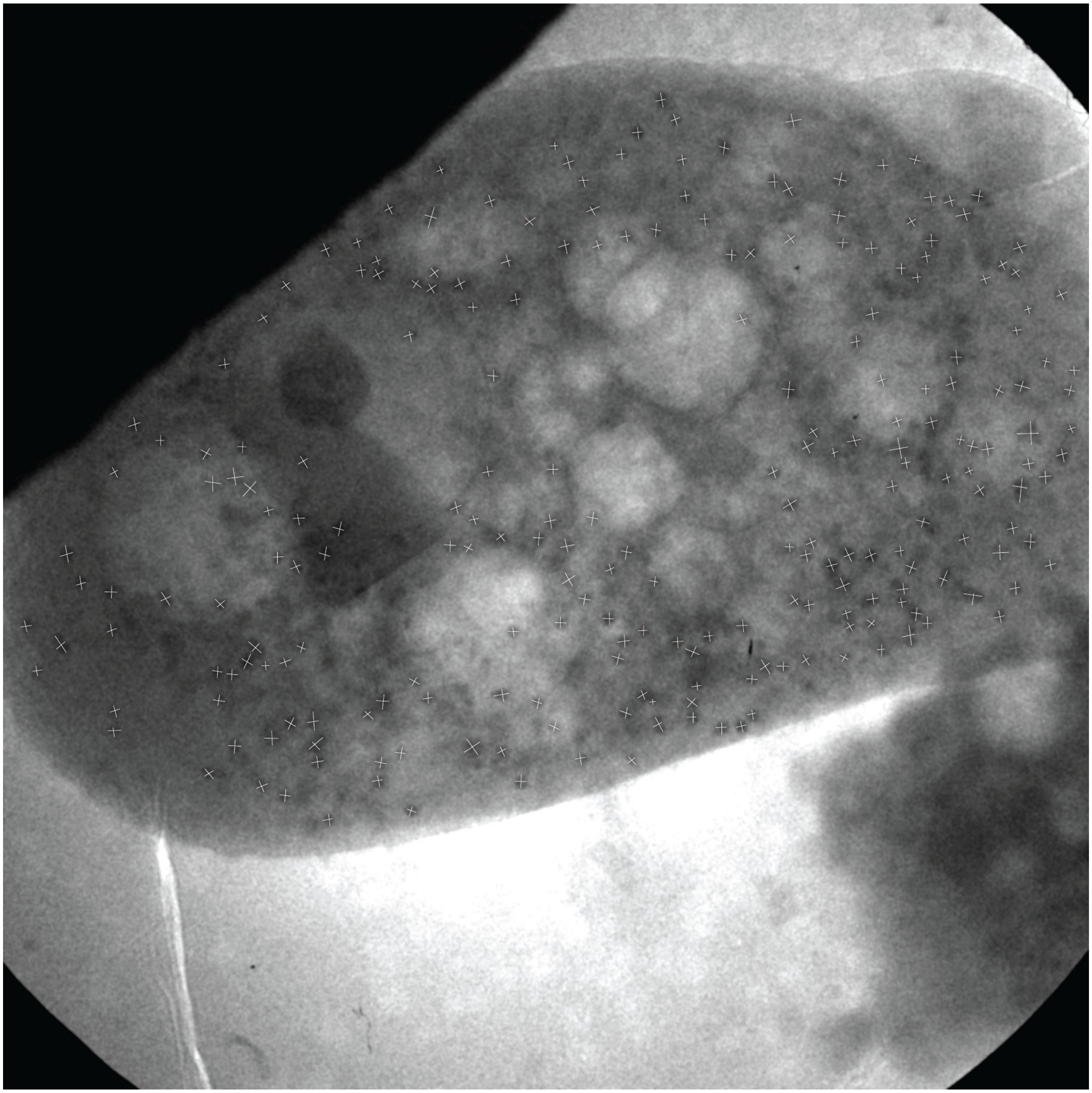

Supplementary Figure S15. Marked structures of the non-infected cell shown in Fig. 2a, resulting in 221 assigned regular cell structures and 5 assigned viral particles.

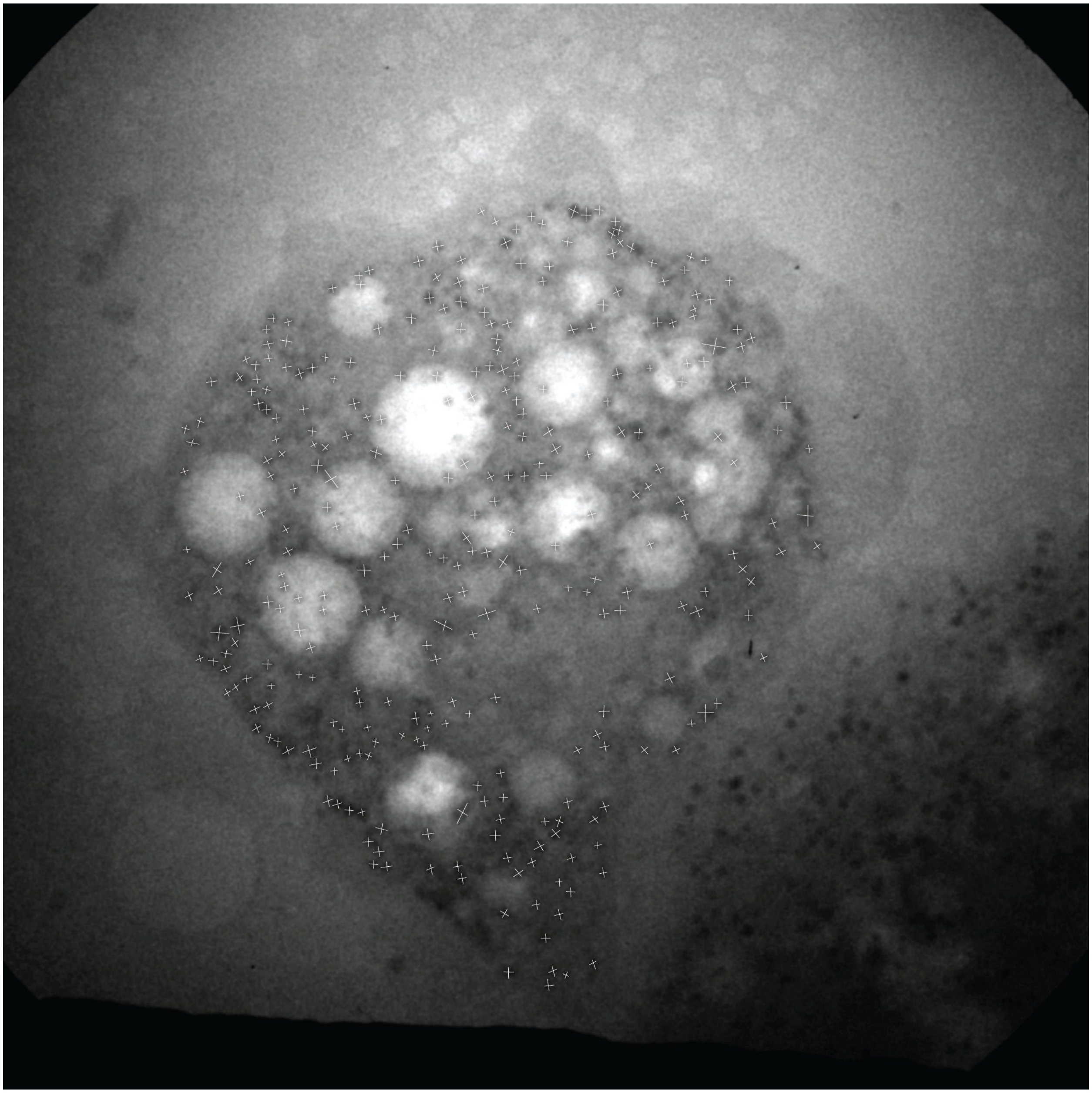

Supplementary Figure S16. Marked structures of the infected cell 12 hpi shown in Fig. 3c, resulting in 308 assigned regular cell structures and 5 assigned viral particles.

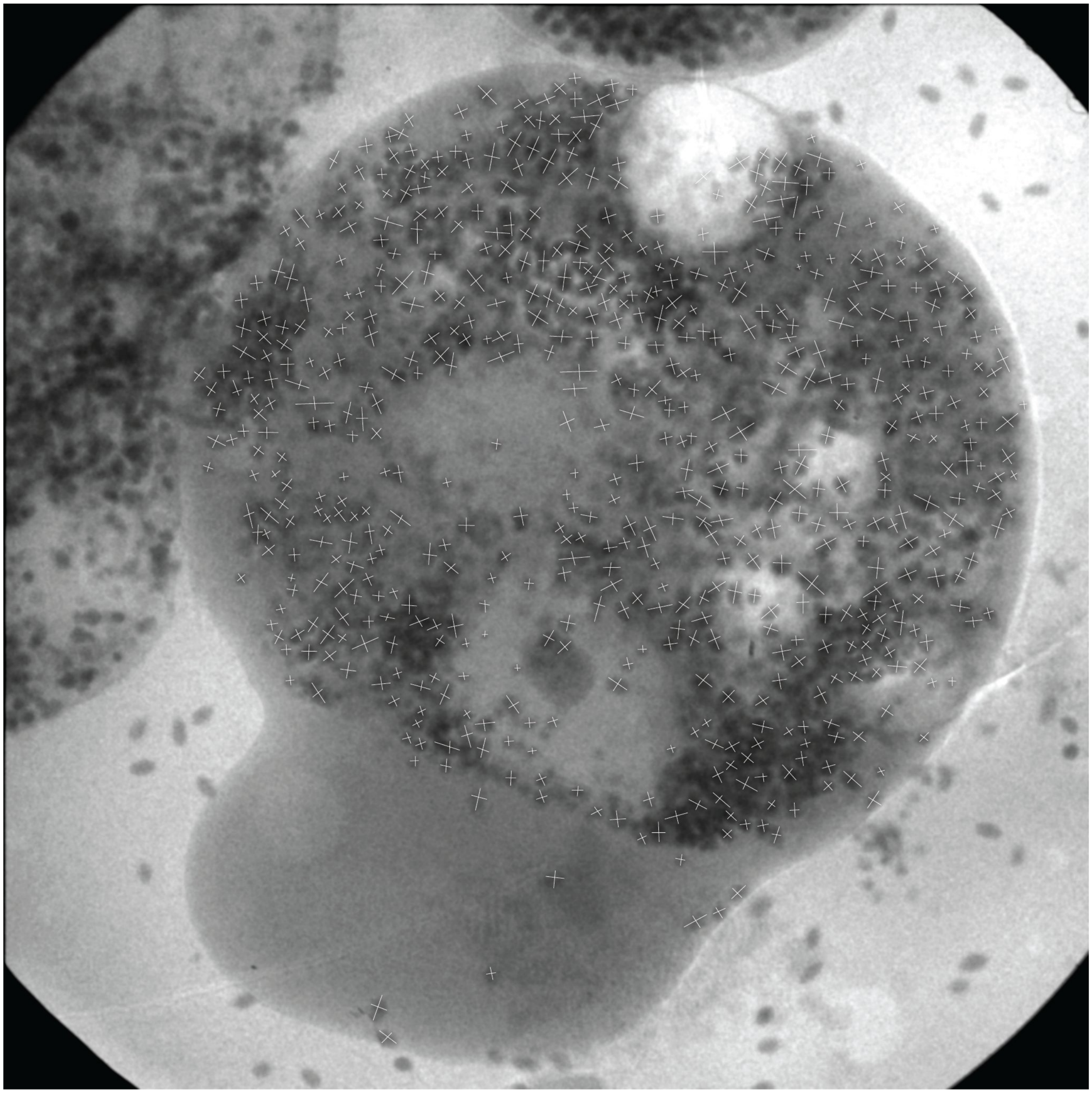

Supplementary Figure S17. Marked structures of the infected cell 54 hpi shown in Fig. 2b, resulting in 434 assigned regular cell structures and 177 assigned viral particles.

C.2. MATLAB scripts to identify viral particles in marked images

```
%========================Run analyze cross image===========================
% Goes through a selected folder containing marked virus images and
% analyzes the content of all images by calling function
%'analyzeCrossImage.m' Then saves info about marked particles and/or
% identified viral particles in a certain folder.
%
% Mikael Kördel, 2018-02-xx
% Last update: 2018-12-11
%==========================================================================

function [ particleInfo, virusInfo] = analyzeCrossImage(file1,minLength,
maxLength, minEllipticity,person)
% Function that finds viral particles in a marked image and extracts
% position, angle, length and width. Calls cross2Ellipse.m to make crosses
% into ellipses.
%
% This version returns the info of both all particles and viral particles,
% respectively.
%
% Mikael Kördel, 2018-08-09
% Last update: 2018-12-xx
%==========================================================================

function [ lengths, widths, xPositions, yPositions, angles ] =
cross2Ellipse( crossImage )
% cross2Ellipse takes an image with black crosses and interprets them as
% the dimension of elliptical particles. Returns the lengths, widths,
% positions and angles of the ellipses. Calls funtion 'crossInfo.m'.
%
% Mikael Kördel, 2018-02-14
% Last uptade: 2018-12-xx
%--------------------------------------------------------------------------

function [ length, width, xPos, yPos , angle] = crossInfo( crossImage )
% crossInfo takes the logical image of a cross and analyzes its length,
% width and position in the xy-plane.  The length (longest dimension)and
% width (shortest dimension) are found using the Radon transform, which
% projects the cross at angles 0:180 degrees (rotAngles) with sub-degree
% steps (angStep).
%
% Mikael Kördel, 2018-02-14
%--------------------------------------------------------------------------
```

C.3. MATLAB scripts to calculate the x-ray absorption in viral particles and cells

```
%==========================Make virus mask=================================
% Load file containing info about viral particles in an image. Draw these
% and make a mask image and save. (The mask image is used by another script
% to calculate the total x-ray absorption in these viral particles.)
%
% Mikael Kördel, 2018-11-30
%==========================================================================



%===============Main Normalization and virus absorption====================
% Fit a 2D gaussian centroid to manually selected points
% Uses lsqcurvefit to fit
% Then normalize cell image using this estimated gaussian illumination.
% Then calculate the accumulated absorption in cell or in marked viral
% particles.
%
% Requires original image, mask image and the illumination values in the
% manually selected points.
%
% NOTE:
%   The initial values in x0 must be close to the correct values in order
%   for the fit to converge.
%
% Mikael Kördel, 2018-11-19
%==========================================================================

function F = D2GaussFunctionRot(x,xdata)
% Returns a 2D Gaussian curve in the points defined by 'xdata', with the
% function parameters defined by 'x'. This 2D Gaussian has two independent
% axes and can have any rotation in the xy-plane. (Rotation angle defines
% by x(6).)
%--------------------------------------------------------------------------
```

D. Supplementary tables

Supplementary Table S1. Number of marked structures in each cell used in the quantitative analysis and assigned by the MATLAB scripts as viral particles. The residual was assumed to be regular cellular structures. For 70 hpi, the marked structures could not accurately be analysed by the MATLAB scripts, which means only particles assumed to be viral particles were marked.

Infection time (hpi)	Number of marked structures	Number of assigned viral particles	Number of assigned regular cellular structures
**non-infected**	221	0	221
**non-infected**	226	5	221
**non-infected**	240	4	236
**non-infected**	519	9	510
**non-infected**	189	1	188
**6**	144	10	134
**6**	415	9	406
**6**	247	2	245
**6**	178	3	175
**6**	166	8	158
**12**	313	5	308
**12**	206	2	204
**12**	272	0	272
**12**	207	6	201
**12**	440	16	424
**12**	377	6	371
**15**	193	8	185
**15**	126	14	112
**15**	209	8	201
**15**	196	12	184
**15**	902	7	895
**18**	318	38	280
**18**	338	55	283
**18**	613	49	564
**18**	403	35	368
**18**	196	28	168
**18**	202	33	169
**21**	228	63	165
**21**	400	43	357
**21**	278	41	237
**21**	415	44	371

**21**	525	53	472
**21**	514	68	446
**24**	270	51	219
**24**	167	55	112
**24**	437	114	323
**24**	525	74	451
**24**	356	56	300
**30**	653	124	529
**30**	380	112	268
**30**	600	114	486
**30**	538	94	444
**54**	550	165	385
**54**	733	304	429
**54**	611	177	434
**54**	507	163	344
**54**	584	227	357
**70**	379	379	only viral particles marked
**70**	294	294	only viral particles marked
**70**	195	195	only viral particles marked
**70**	235	235	only viral particles marked

E. References

1. G. Mahmoudabadi, R. Milo, R. Phillips, Energetic cost of building a virus. *Proc. Natl. Acad. Sci. USA* **114**, E4324–E4333 (2017).
2. M. Lynch, G. K. Marinov, The bioenergetic costs of a gene. *Proc. Natl. Acad. Sci. USA* **112**, 15690–15695 (2015).
3. R. Siddiqui, N. A. Khan, Biology and pathogenesis of Acanthamoeba. *Parasites and Vectors*. **5**, 6 (2012).
